# On the Effect of Melittin on Surface Properties of Erythrocyte and Mitochondrial Membranes

**DOI:** 10.3390/membranes16010011

**Published:** 2025-12-31

**Authors:** Virjinia Doltchinkova, Victoria Vitkova, Meglena Kitanova, Milena Shkodrova, Siya Lozanova, Avgust Ivanov, Chavdar Roumenin

**Affiliations:** 1 Institute of Robotics “Saint Apostle and Gospeller Mathew”, Bulgarian Academy of Sciences (IR-BAS), Acad. Georgi Bonchev Str., Bl. 2, P.O. Box 79, 1113 Sofia, Bulgaria; lozanovasi@abv.bg (S.L.); avgust.ivanov@abv.bg (A.I.); roumenin@abv.bg (C.R.); 2 Soft Matter Physics Department, Institute of Solid State Physics, Bulgarian Academy of Sciences, 72 Tsarigradsko Chaussee Blvd., 1784 Sofia, Bulgaria; 3 Department of Genetics, Faculty of Biology, Sofia University “St. Kliment Ohridski”, 8 Dragan Tzankov Blvd., 1164 Sofia, Bulgaria; m.kitanova@uni-sofia.bg; 4 Laboratory of Bioenergetics, Department of Animal and Human Physiology, Faculty of Biology, Sofia University “St. Kliment Ohridski”, 8 Dragan Tzankov Blvd., 1164 Sofia, Bulgaria; milshk@abv.bg; 5 Center of Competence in Personalized Medicine, 3D and Telemedicine, Robotic-Assisted and Minimally Invasive Surgery, Medical University of Pleven (MU-Pleven), 5800 Pleven, Bulgaria

**Keywords:** biological membranes, surface properties, zeta potential, hemolysis, lipid peroxidation, fluorescence microscopy, membrane transport

## Abstract

Many biomedical applications require a detailed understanding of the action of antimicrobial peptides on biological membranes. The cationic hemolytic peptide melittin, a major component of European honey bee (*Apis mellifera*) venom, is considered a model for elucidating lipid–protein interactions that are important for the function of biological systems. Here, we address the surface properties of human erythrocytes and rat liver mitochondrial membranes under in vitro melittin treatment. These membranes are negatively charged at neutral pH and represent primary targets of melittin’s effects in the onset of inflammatory diseases. The correlation between the functional activity of membrane systems and their surface electrical charge was assessed using microelectrophoresis, hemolysis assays, membrane transport measurements, lipid peroxidation analysis, and fluorescence microscopy. A mechanistic hypothesis for the divergent effects of sub-lytic, pre-pore doses of melittin on erythrocytes and mitochondria is discussed. At low concentrations, melittin interacts electrostatically with erythrocyte membranes, resulting in altered proton transport through the Band 3 protein. Melittin also induces changes in erythrocyte morphology and malondialdehyde content, as well as aggregation of mitochondrial vesicles. The electrokinetic mechanism of melittin action, associated with membrane stability, provides a novel perspective on its potential relevance to biomedical applications.

## 1. Introduction

Investigations on the interaction between biological membranes, lipid bilayers and peptides are essential for understanding the insertion processes of membrane proteins, as well as the mechanisms of action of antimicrobial peptides and toxins [1,2,3,4,5,6,7,8,9,10]. Further insight into the mechanisms by which surface-active peptides, known as antimicrobial peptides (AMPs), interact with membranes is required to understand the electrokinetic stability of AMP-associated biological structures. 

Biochemical and biophysical methods, along with biological model systems, have been widely used for several decades to investigate the structure and properties of melittin from the European bee, which has attracted considerable interest for its potential therapeutic applications [11].

Melittin consists of 26 amino acid residues and accounts for ~50% of the dry weight of bee venom [12,13]. Its primary structure is NH_2_-(+)Gly-Ile-Gly-Ala-Val-Leu-Lys(+)-Val-Leu-Thr-Thr-Gly-Leu-Pro-Ala-Leu-Ile-Ser-Trp-Ile-Lys(+)-Arg(+)-Lys(+)-Arg(+)-Gln-Gln-CONH_2_ [14,15]. Melittin binds to the polar head groups of phosphatidylserine via intermolecular ionic, ion-polar and hydrogen bonds involving amino acid residues of eight amino acids—T11, G12, P14, S18, K21, R22, Q25, Q26—on the molecular surface of melittin molecule [16]. The binding affinity of melittin to phosphatidylcholine, phasphatidylserine, phosphatidic acid and cardiolipin, as well as the localization of phospholipid-binding sites on the surface of melittin, has been examined using biophysical methods [17]. In vitro studies of erythrocyte and mitochondrial membranes may provide a clear interpretation of the results, thereby revealing the physicochemical mechanisms underlying melittin’s in vivo action. Antimicrobial peptides are extensively investigated in relation to challenges associated with drug-resistant bacteria [18]. Elucidating the biophysical properties of erythrocytes and mitochondria interacting with melittin provides important insights into its membrane-targeting mechanisms.

The surface electrical charge of the membrane is a key characteristic in cellular metabolism [19]. It plays an important role in cell signaling and can significantly influence many signaling molecules involved in cancer biology. Surface charge affects ion exchange between cells and the extracellular environment and can be quantified by zeta potential measurements. Zeta potential provides information on the surface properties of cells, subcellular structures, and inorganic materials, and serves as a key parameter for assessing the stability of these systems [20]. Various diseases (inflammatory diseases, leukemia, hemolytic anemias, renal failure, etc.) lead to alterations in membrane surface charge, which in turn promote agglutination processes and impair blood microcirculation. The increased propensity for erythrocyte aggregation observed in sepsis and other pathological conditions is attributed to a reduction in erythrocyte surface charge density and a consequent shift in the balance of aggregation forces, resulting from decreased electrostatic repulsion between neighboring cells [21]. The main components of the erythrocyte membrane surface—sialic and hyaluronic acid—determine the sign and magnitude of the red blood cell surface charge with sialic acids playing a particularly significant role in tumor metastasis [22]. The electrophoretic mobility of erythrocytes is genetically determined and exhibits species-specific characteristics. Electrophoretic mobility provides information on the dynamics of membrane surface charge by allowing the determination of the zeta potential or subcellular structures. The significance of zeta potential, which influences both cell–cell and cell–membrane interactions, has been thoroughly detailed by Hughes [23]. Naturally occurring changes in red blood cell structure and physiological function during aging are associated with corresponding alterations in the viscoelastic properties and electrophoretic behavior of their membrane surfaces [23,24].

Melittin is a cationic hemolytic peptide, a major component of the toxin of the European honey bee, *Apis mellifera*. It is widely used as a simplified model for studying lipid–protein interactions that are critical for the function of other biological systems [25,26]. It is well established that melittin can interact with membrane proteins through electrostatic forces [27,28,29].

Changes in the energetic state of mitochondria, including both energization and de-energization, are known to alter the electrostatic characteristics of their membranes [30,31,32]. When mitochondria are energized, their electrokinetic properties are significantly influenced by changes in the surface charge of the inner membrane [30,32]. Given the limited amount of data, the study of the electrokinetic properties of mitochondrial membranes is relevant for analyzing the electrostatic effects of melittin. Melittin induces inverted or concave lipid structures in natural membranes, leading to destabilization of the bilayer [33]. 

Erythrocyte and mitochondrial membranes are negatively charged at neutral pH of the suspending medium [24,34,35]. Although less specialized than other cell membranes, they have functions that are representative of the plasma membrane. 

The erythrocyte membrane exhibits an asymmetric phospholipid distribution. The outer lipid bilayer is mainly composed of sphingomyelin and phosphatidylcholine, while the inner layer contains mainly phosphatidylserine and phosphatidylethanolamine [36,37]. The asymmetric composition of biological membranes leads to a different response to exogenously imported molecules and various functional consequences, including changes in the shape of intact cells [38]. Higher capacitance of egg phosphatidylcholine bilayers in the presence of melittin (0.10 g/L) as a consequence of bilayer thinning and alterations in the dielectric permittivity of melittin-treated membranes was reported [24].

Mitochondria possess outer and inner membranes [39]. The outer mitochondrial membrane is rich in lipids and cholesterol, but almost no cardiolipin is encountered. The outer membrane is negatively charged due to the lipid composition and partly due to the protein component. In liver mitochondria, 5% of the proteins are located in the outer membrane. They are represented by porin, a translocator protein complex (TOM) and many enzymes. 

The model systems with different geometries studied here (erythrocytes and mitochondria) have specificity in their lipid matrices, which are the main target of melittin action.

It has been experimentally shown that there were about 1.8 × 10^7^ binding sites for melittin per erythrocytes, which bind to lipids and not to specific receptors [6]. Lipid composition also affects the extent of membrane lysis. Negatively charged lipids reduce the lytic activity of melittin. Cholesterol, phosphatidylglycerol and phosphatidylserine and plant phospholipids such as monogalactosyldiacylglycerol and digalactosyldiacylglycerol reduce the lytic activity of melittin. This effect is likely due to ionic interactions of melittin with the negatively charged lipid head groups. Additionally, hydrogen bonds can form between melittin and the hydroxyl groups of sugar residues, as observed with monogalactosyldiacylglycerol [40,41].

Melittin interacts differently with various types of lipids in biological membranes. Its affinity for membranes composed of negatively charged lipids is approximately 100 times higher than for membranes composed of zwitterionic lipids [32]. A pronounced electrostatic component characterizes the interaction of melittin with membranes. Upon attachment to POPC bilayers, melittin adopts a “U-shaped” conformation leading to the formation of defects and reorientation of the lipid head groups [42]. Melittin is a relatively short protein with α-helical conformation in the membrane. At higher concentrations, melittin forms aggregates and induces pore formation in the membrane [5,43].

The effect of melittin on mitochondria remains insufficiently understood, although its similarity to mitochondrial protein signal sequences has been recognized for years. In this study, we investigate changes in the electrophoretic mobility of mitochondrial membranes following melittin treatment. Based on the Gouy–Chapman’s theory, which describes interactions between charges and the electrostatic contribution to total binding energies, the effective charge of melittin was calculated to be approximately +2 at erythrocytes (Z_eff_ = 2.2) and mitochondria (Z_eff_ = 2.3) [44,45].

Certain diseases, such as hypertension, hemoglobinopathies, lead to changes in erythrocyte shape and aggregation capacity. Our study focused on measurements of erythrocyte electrophoretic mobility in the presence of exogenously added melittin, which revealed changes in cell shape and electrokinetic potential even during in vivo aging. In contrast, the functional activity of mitochondria following melittin treatment was altered differently during in vitro aging reflecting the electrokinetic stability of these subcellular structures.

## 2. Materials and Methods

### 2.1. Materials

All chemicals used in the present study were of analytical grade. Melittin from honey bee venom (65–85% minimum purity (HPLC), phospholipase A_2_ ≤ 2% units/mg solid; M-7391, Sigma-Aldrich; phospholipase A2 component could potentially influence the results of the present study, even at the low concentrations used); Trizma^®^hydrochloride, Tris [hydroxymethyl] aminomethan hydrochloride, Sigma Aldrich, Inc., St. Louis, MO, USA; Na_2_HPO_4_, sodium phosphate dibasic; KH_2_PO_4_, potassium phosphate monobasic; Concanavalin A from *Canavalia ensiformis* (Jack bean); FITC (fluorescein isothiocyanate) conjugate; extent of labeling: 3–6 mol FITC per mol protein; Sorbitol ((2S,3R,4R,5R)-Hexane-1,2,3,4,5,6-hexol); Hepes-N-(2-Hydroxyethyl)piperazine-N’-(2-ethanesulfonic acid); sodium bicarbonate (NaHCO_3_), NaCl, KCl, Sodium hydroxide (NaOH) were purchased from Sigma-Aldrich (St. Louis, MO, USA), and the chemicals were as follows: NaCl, KCl, CaCl_2_ (Merck), EDTA (Ethylenediaminetetraacetic acid, EDTA disodium salt), TCA (trichloroacetic acid); 2-thiobarbituric acid (TBA); NaN_3_, sodium azide, hydrated Copper (II) sulphate (CuSO_4_× 5H_2_O), potassium sodium tartrate (KNaC_4_H_4_O_6_× 4H_2_O) were purchased from Sigma-Aldrich (St. Louis, MO, USA). Propidium iodide was purchased from Vector Laboratories Inc., Newark, CA, USA. D(+)-Saccharose was purchased from Riedel De Haën AG, Seelze, Hannover, Germany; 2,4-DNP (2,4-dinitrophenol, 20% H_2_O). Oxytocin-Richter 5 I.U./mL, injection solution was purchased from SOpharmacy, Bulgaria. Bidistilled water from a quartz distiller was used. All buffer media solutions were previously filtered through Whatman^®^ membrane filters PTFE, cellulose nitrate, pore size 0.2 µm, diam. 47 mm, non-sterile, Whatman 7402-004, Whatman Article No. 28420767, Sigma Aldrich, Merck KGaA, Darmstadt, Germany. Melittin was injected into the erythrocytes or mitochondria from a stock solution in double distilled water (mg/mL Melittin = 0.35 µM Mt) so that the studied nanomolar concentration range of Mt in phosphate-buffered saline (PBS), pH 7.4 or saline sorbitol buffer (SSB), pH 7.2, respectively, was used. All treated preparations were stored at 4 °C until use.

### 2.2. Isolation of Biological Membranes

#### 2.2.1. Erythrocyte Preparations

Erythrocytes were prepared from citrate-containing blood bank of phenotype A (Rh+); AB (Rh+); O (Rh+) supplied by National Centre of Hematology and Perfusion in Sofia. The present study complied with the ethical regulations and legislation in European Union and Bulgaria. The experiments were performed in compliance with the World Medical Declaration of Helsinki [46] (https://www.wma.net/policies-post/wma-declaration-of-helsinki/ (accessed on 31 October 2008). Ethics Committee of Sofia University “St. Kliment Ohridski” permission, approval codes, RD-04-68, 2 February 2023, was used. Blood samples were collected from blood bank. The blood banks of phenotypes A (Rh+):5 bags (containers), AB (Rh+):3 bags (containers), O (Rh+):4 bags (containers) were used for the biophysical experiments. Experimental data were analyzed per donor. According to the research data, the electrophoretic mobility of erythrocytes was not affected by the group and rhesus affiliation of the blood, gender or race of the donor. Washing of erythrocytes did not affect the electrokinetic properties [47]. Erythrocytes were isolated after triplicate centrifugation with isotonic phosphate-buffered saline (PBS), pH 7.4, in a microcentrifuge MIKRO 22R, *Hettich* (Kirchlengern, Germany) at 1200× *g* for 5 min with phosphate-buffered saline (PBS, pH 7.4) and suspended in the same buffer for microelectrophoresis measurements [24,48].

#### 2.2.2. Isolation of Mitochondria

Intact liver mitochondria were isolated from male albino rats as in [49,50] with modification precisely noticed in [51]. The lab rats (*Rattus norvegicus*) (Wistar strain; weight 120–150 g) were supplied from the Sofia University “St. Kliment Ohridski” breeding base under standard conditions (temperature 22 ± 2 °C, 12 h light/dark schedule, standard food and water ad libitum [51]. One animal was used for the isolation of mitochondria, and seven rats were used to carry out the assigned biophysical tasks. The present study complied with the ethical regulations and legislation in both European Union and Bulgaria. Studies were performed in accordance with the institutional ethical guidelines in compliance with “Directive 2010/63/EU of the European Parliament and of the Council of 22 September 2010 on the protection of animals used for scientific purposes” [52]. The liver was washed in a cold isolation medium containing 250 mM sucrose and 1 mM EDTA-KOH (pH 7.5). The final mitochondrial pellet was suspended in 1–1.5 mL suspending medium. Mitochondria were coupled in control assay by uncoupling of oxidative phosphorylation with 2, 4-Dinitrophenol (DNP) [53].

### 2.3. Hemolysis

We used hemolysis test (Hb-release) at different concentrations of NaCl to determine the degree of hemolysis. The erythrocyte hemolysis test was fixed [54] by adding erythrocytes to a series of hypotonic solutions with decreasing NaCl concentrations (0.9–0.3% NaCl) at 5% Hct, with incubation for 30 min at 25 °C with gentle mixing (speed of 300 rpm using TMix Thermal mixer, Analytic Jena AG, Jena, Germany). Determination of hematocrit (Hct) was described as in [24]. Afterward, 1.5 mL of the samples with different salt concentrations were diluted with the samples of the erythrocyte suspensions previously incubated with melittin (1 h at 37 °C) at 25 °C for 30 min. The erythrocytes incubated with different hypotonic solutions were then centrifuged (12,000× *g* for 1 min), and after that, the supernatants were removed. The hemoglobin of each supernatant was measured from the absorbance at λ = 576 nm immediately after centrifugation [55] using a BOECO Spectrophotometer S-200 (VIS), Boeckel + Co (GmbH + Co) KG, Hamburg, Germany. The Abs (absorbance) was calculated by plotting the relationship between the absorbance at 576 nm, respectively, versus the absorbance at the appropriate concentration of the NaCl solution. The value of Δ*Hemolysis* (Hb–release) was calculated as follows:

(1) $$∆Hemolysis=\frac{\left({Abs}_{treatment,\;x}-{Abs}_{treatment,0.9\%\;NaCl}\right)}{{Abs}_{treatment,x\;}}$$

where ${Abs}_{treatment,\;x}$ is the absorbance *Abs* (λ = 576 nm) of the release of hemoglobin from erythrocytes in the presence or absence of different concentrations of melittin, measured at λ = 576 nm and ${Abs}_{treatment,0.9\%\;NaCl}$ is the absorbance from the release of hemoglobin in erythrocytes in the presence or absence of melittin, suspended in 0.9% NaCl solution, respectively.

#### 2.3.1. Osmotic Fragility

To account for the rate of osmotic hemolysis, monitoring is also required at 0.5% NaCl. Osmotic fragility of erythrocytes was characterized by the hemolysis of these cells to the action of destructive agents—chemical, osmotic, mechanical, thermal. Osmotic fragility of erythrocytes was of clinical importance in the diagnosis of hemolytic anemias, with changes in hemolytic crises being observed. The hemolysis rate parameter was related to the process of pore formation in the erythrocyte membrane, which occurred when a hemolytic critical volume was reached. This parameter did not depend on the output volume of the cell and provided information about the final stage of reactions related to cell disintegration when an osmotic gradient was applied. It was believed that with an increase in the rate of hemolysis, the fluidity of the membrane should also increase. The influence of membrane modifiers, like bee venom melittin, could be associated with corresponding changes in the fluidity of the erythrocyte membrane.

The value of ∆Hemolysis was calculated as follows:

(2) $$∆Hemolysis=\frac{\left({Abs}_{treatment,0.5\%\;NaCl}-{Abs}_{treatment,x}\right)}{{Abs}_{treatment,0.5\%\;NaCl}}$$

where ${Abs}_{treatment,x\;\;}$ is the absorbance *Abs* (λ = 690 nm) of the release of hemoglobin from erythrocytes with or without different concentrations of melittin, measured at 690 nm and ${Abs}_{treatment,0.5\%\;NaCl}\;$, is the absorbance from the release of hemoglobin in erythrocytes without melittin, suspended in 0.5% NaCl solution, respectively.

Determination of the rate of osmotic hemolysis of erythrocytes with or without Mt was carried out using a BOECO spectrophotometer. The extinction of the erythrocyte suspension (10 µL in 2 mL solution) at a wavelength of λ = 690 nm every 10 s for 2 min was reported. The rate of hemolysis of erythrocytes was determined when they were placed in 0.5% NaCl. Each measurement was repeated three times, and the corresponding values were averaged. The percentage change in absorbance was calculated according to the following formula:

(3) $$k=\frac{2.303}{t}lg\frac{\left({Abs}_{0\;}\right)}{{Abs}_{\;x\;}}$$

where t is the time from the moment of entry to the moment of registration (s); ${\;Abs}_{0\;}$ is the value of untreated erythrocytes at 0.5% NaCl;${\;Abs}_{\;x\;}$ is the absorbance parameter with different concentrations of melittin and reading time. The rate constants at 20, 40 and 70 s noted by k_10–20_, k_20–40_, or k_40–70_, respectively, were calculated.

#### 2.3.2. Acid-Induced Hemolysis Assay

The acid-induced hemolysis of human erythrocytes was studied in isosmotic media (8.5% NaCl) in the presence of 0.004 N HCl as a hemolytic agent. Erythrograms were obtained according to the classical method [24,56,57]. The difference between the Abs of erythrocyte membranes at the beginning of the process of hemolysis and the final value of Abs represented the change in absorbance coefficient and we approximated its value as 100 percent. The changes between two following Abs values were represented in percentage of the whole change in absorbance. The upper values were proportional to the average velocity of the hemolytic process during every separate interval of 30 s between 2 following measurements. The dependence of acid-induced hemolysis over time for 10 min was represented by erythrogram. Consequently, the acid erythrogram representing the first derivative of optical density kinetic curve and characteristics of erythrocytes population heterogeneity was used [56,57]. Acidic fragility measurements in erythrocytes represented the method for investigations and diagnosis in medicine. It was used to study the mechanism of pathological processes under the action of several drugs or biologically active compounds [24,58].

### 2.4. Microelectrophoretic Studies

Electrophoretic mobility studies were performed using a Cytopherometer (OPTON, Feintechnik Ges.m.b.H., Zeiss–OPTON, Oberkochen, Germany). The value of the EPM of erythrocytes was expressed in units of 10^−8^ m^2^V^−1^s^−1^. Values represented the mean of three independent preparations (45–105 cells). The time for the movement of each cell over a distance of 16 µm was recorded, depending on the mobility of the cells, bidirectionally when changing the electric field. Each measured sample included three consecutive readings of 15–35 cells. Each value of the biophysical measurements was the mean ± SD of the three independent preparations (n = 3), with each of them averaged over three repetitions. Electrophoretic studies were conducted at a temperature of 25 °C and a direct current of 5 mA. The SDs were between 3.7 and 12% for erythrocytes. The zeta potential (*ζ*) was calculated from the electrophoretic mobility [24,48,59]:

(4) $$\zeta \;=\frac{\eta u}{\varepsilon_{r}\varepsilon_{0}}$$

where ζ is in units of mV, $\varepsilon_{r}\;=\;78.5\;\left(at\;25{}^{\circ}C\right)$ is the relative dielectric permittivity of the aqueous phase, $\varepsilon_{0}\;=\;8.8542\;\cdot 10^{-12}Fm^{-1}$ is the permittivity of free space and η = 0.001393 Pa.s is the viscosity of the phosphate-buffered solution—PBS: 136.9 mM NaCl, phosphate buffer (10.1 mM Na_2_HPO_4_, 1.8 mM KH_2_PO_4_), pH 7.4 at 25 °C as in [60]. The surface electrical charge of erythrocytes suspended in isotonic ionic strength buffer ($I=0.145\;M^{-1}$) is expressed in C/m^2^ [60].

Electrophoretic mobility (EPM) of intact mitochondria was measured by microelectrophoresis (OPTON Cytopherometer, Feintechnik Ges.m.b.H., Zeiss–OPTON, Oberkochen, Germany) using a rectangular cell and platinum electrodes. Electrophoretic migrations were determined for both forward and backward runs over 32 μm at 4 mA direct current. The value of the EPM of intact mitochondria suspended in saline sorbitol buffer (4.5% sorbitol, 14.50 mM NaCl, 0.6 mM NaHCO_3_, pH 7.2 at 25 °C), buffer with ionic strength of (I=0.0151 M), was expressed in units of 10^−8^ m^2^V^−1^s^−1^. The zeta potential (*ζ*) was calculated from the electrophoretic mobility, *u*, using Henry’s relation [59] as in [61]:

(5) $$\zeta =\frac{3\;u\eta }{2\;\varepsilon f(ka)}$$

where *ζ* is zeta potential in units of mV, u the electrophoretic mobility of intact mitochondria expressed in units of 10^−8^ m^2^V^−1^s^−1^, η is the viscosity of SSB (η = 0.00133 Pa.s), ε is the dielectric constant (*ε* = *ε_r_ε_o_*, where *ε_r_* = 78.5 (at 25 °C) is the relative dielectric permittivity of the aqueous phase, *ε_o_* = 8.8542 × 10^−12^ Fm^−1^ is the permittivity of free space) and f(ka) the Henry’s function $f\left(ka\right)=1\;$ [61]. Bee venom melittin was injected immediately after suspending of mitochondria to the buffer, and the EPM of mitochondrial vesicles in an electric field was measured. Values represented the meaning of three replications (48–108 vesicles). All experiments were performed in triplicate. The SDs were between 4 and 11% for mitochondria.

The surface electrical charge at the surface of the membrane was calculated by [60] and was expressed in C/m^2^:

(6) $$\frac{136.6\sigma }{\surd C}=sin\;h\left(\frac{z\psi_{0}}{51.38}\right),$$

where $\psi_{0}$ is in mV, A=136.6 at 25 °C, $A=1/\surd \left(8N\varepsilon_{r}\varepsilon_{0}kT\right)$, N=6.022×10^23^ mol^−1^ is the Avogadro constant.

The values of surface charge were calculated based on the assumption that $\zeta ≅\psi_{0\;}$ [60]. Zeta potential values characterized the electrokinetic stability of erythrocytes or mitochondria [60,61].

### 2.5. Electrostatic Free Energy Determinations

The electrostatic coulombic part of the electrical work performed in charging up the surface of erythrocytes or mitochondria ($G_{el}^{s}$) was calculated [51,62,63]. The electrostatic free energy of erythrocytes or mitochondrial vesicles upon melittin treatment was determined [62]. The Debye–Hückel length for erythrocytes ($\frac{1}{k}≅0.80\;nm$) or for mitochondria ($\frac{1}{k}≅24.74\;nm$) at 25 °C was observed [64].

### 2.6. Measurements of Proton Transport and Conductivity

The experimental study on anion–proton co-transport based on the measurement of net proton flows was associated with erythrocyte Band 3-mediated net anion transfer. Erythrocyte suspension (500 µL, Hct = 23%) with or without melittin was added to isotonic sucrose medium and the time course of the pH of the medium was registered for 10 min. The changes in pH were presented in percentage of control. All experiments were carried out at 25 °C as described in [24,48,65].

Proton efflux and conductivity began by mixing 500 µL of erythrocytes suspended in 50 mL of 0.3 M sucrose (NaOH), pH 7.4. The pH and conductivity of the erythrocyte suspension with or without different concentrations of melittin at the same time were measured using a Thermo Fisher Scientific CyberScan PC 510 (Oakton® Instruments Pte Ltd., Singapore) (USA/Singapore) pH/conductivity meter.

The results for the proton efflux alteration in extracellular media in the presence of different concentrations of melittin were obtained from membrane transport measurements in erythrocyte suspending media every 20 s for 10 min with gentle mixing.

The value of ∆pH(%) was calculated as follows:

(7) $$∆pH=\frac{\left({pH}_{o}-{pH}_{t}\right)}{{pH}_{0}}\cdot \;100$$

where ${pH}_{o}$ and ${pH}_{t}$ are the pH values of erythrocytes in melittin-free medium and in the presence of Mt molecules concentration, respectively.

### 2.7. Detection of Malondialdehyde (MDA) Assay

MDA assay with erythrocytes was evaluated by the analysis of thiobarbituric acid reactive substances (TBARS) with modifications according to [66,67] and as described in [49]. The erythrocyte suspensions (500 μL erythrocytes in PBS, pH 7.4, 2 mM NaN_3_, Hct = 23%) with or without melittin after incubation were homogenized in 400 μL of 28% trichloroacetic acid (TCA) and centrifuged at 12,500× *g* for 4 min. After centrifugation, 1 mL of supernatant was mixed with 500 μL of 1% thiobarbituric acid (TBA) in 1% NaOH, and the mixture incubated in boiling water for 30 min. The suspending medium was centrifuged at 12,500× *g* for 2 min, and the absorbance of λ = 532 nm was measured by means of an BOECO Spectrophotometer S-200 (VIS), Boeckel & Co. (GmbH & Co. KG, Hamburg, Germany) to determine the MDA content. The lipid peroxidation of erythrocytes samples without melittin treatment represented the control values. There was a second positive control of measurements of erythrocytes in the presence of 50 mM H_2_O_2_, where the maximal content of TBARS products was observed. TBARS molar concentrations, *c*, were calculated as follows:

(8) $$c=\frac{Abs}{\varepsilon l},$$

where Abs is the absorbance, ε stands for the molar extinction coefficient at 532 nm ($\varepsilon_{532}=154,000\;M^{-1}{\mathrm{cm}}^{-1}$) and l represents the optical path length.

Malondialdehyde (MDA) content of mitochondria was measured according to [68] with slight modifications [51]. Mitochondria (1 mg protein/mL) were incubated in the dark at 37 °C for 60 min with or without different concentrations of melittin in physiological ionic strength and pH. The buffer (KCl-Tris·HCl) was composed of 150 mM KCl, 50 mM Tris·HCl, pH 7.4 (1 mL final volume). Mitochondria were treated by melittin for 1 h at 37 °C, with a mixing speed of 300 rpm (TMix Thermalmixer, Analytic Jena AG, Jena, Germany). The mitochondrial suspensions with or without melittin doses were mixed with 750 μL of 28% trichloroacetic acid (TCA) and the precipitated proteins were removed by centrifugation at 12,500× *g* for 2 min. After centrifugation, 0.5 mL of supernatant was mixed with 2 mL of 1% (*w*/*v*) 2-thiobarbituric acid (TBA) in 20% NaOH, and the mixture incubated in boiling water for 30 min. Afterwards the reaction was stopped by cooling the samples at room temperature. The 2-thiobarbiturate–malonaldehyde adduct was quantified spectrophotometrically at 533 nm by means of a BOECO Spectrophotometer S-200 (Germany) to determine the MDA content. The mitochondrial control with 50 mM H_2_O_2_ was also prepared. TBARS molar concentrations were calculated using Equation (8) with molar extinction coefficient $\varepsilon_{533}=153,000\;M^{-1}{\mathrm{cm}}^{-1}$. Afterwards the reaction was stopped by cooling the samples in an ice bath. MDA reacted in the TBA test to generate a colored product. In acid solution the product absorbed light at 533 nm. The lipid peroxidation of mitochondrial membranes was determined by production of thiobarbituric acid reactive substances (TBARS) and expressed in μmol L^−1^ [66,67,68].

### 2.8. Fluorescence Microscopy

#### 2.8.1. Fluorescence Microscopy of Erythrocytes

FITC-concanavalin A (FITC-CoA) (MW 102,000 Da) labeling solutions were dissolved in distilled water at 2 mg/mL as a stock solution in dark tubes. Concanavalin A-FITC-labeled from *Canavalia ensiform* is (Jack bean) Type IV, FITC content of 3.6 mol/mol lectin had an affinity for terminal-D-mannosyl and-D-glycosyl residues. The samples were diluted with PBS, pH 7.4, to final concentration of 40 µg/mL before use. Fluorescence microscopy of erythrocytes with FITC-labeled CoA samples followed the protocol described in [24]. For better stabilization of the fluorescent signal in erythrocytes with or without bee venom melittin, they were allowed to stain better for the formation of a luminescent halo around the erythrocyte, which did not form immediately after incubation with FITC-concanavalin A. The cells were faintly stained, and their luminescence was not immediately noticeable after treating them with FITC-labeled lectin on the same day after incubation following the protocol for labeling them. The results were documented with an Axiocam 202 mono digital camera (Carl Zeiss Microscopy GmbH, Jena, Germany) and ZEN 2.5 (blue edition) software (Carl Zeiss Microscopy GmbH, Jena, Germany) and processed with Adobe Photoshop CC 2021 software [69].

#### 2.8.2. Fluorescence Microscopy of Rat Liver Mitochondria

The samples were observed on a Zeiss Axioscope 5 with LED Illumination Colibri 3 epifluorescence microscope at 1000× magnification (objectives 100× and eyepieces 10×) under immersion, with appropriate excitation filters for propidium iodide (PI) staining solution and fluorescein isothiocyanate (FITC): (blue, 478–495 nm). The images were documented with an Axiocam 202 mono digital camera (Carl Zeiss Microscopy GmbH, Jena, Germany) and ZEN 2.5 (blue edition) (Carl Zeiss Microscopy GmbH, Jena, Germany), (blue edition) software [69]. FITC-*concanavalin A* (MW 102,000 Da) labeling was dissolved in bidistilled water at 2 mg/mL as stock solution in dark tubes.

It was diluted with Hepes-buffered saline (HBS: 5 mM Hepes, 2.7 mM KCl, 146 mM NaCl, pH 7.4 (NaOH)) [70] to 40 µg/mL before use for fluorescence microscopy of mitochondria under melittin treatment. Mitochondria were incubated with or without melittin doses for 60 min at 37 °C in dark, mixing speed of 300 rpm (Thermal mixer TMix, Analytic Jena AG, Jena, Germany) as described [51].

Results were documented with an Axiocam 202 mono digital camera (Carl Zeiss Microscopy GmbH, Jena, Germany) and ZEN 2.5 (blue edition) software (Carl Zeiss Microscopy GmbH, Jena, Germany) (ZEISS ZEN (Blue edition)) and processed with Adobe Photoshop CC 2021 software [69].

### 2.9. Statistical Analysis

The data were averaged from triplicate measurements for every sample and expressed as weighted mean ± SD. The significant means were determined by use of one-way ANOVA using SigmaStat and MiniTab v.17 statistical software. One-way ANOVA was performed with Dunn’s Test or the Student–Newman–Keuls method taking *p* < 0.050 as significant and *p* < 0.001 as highly significant and denoted by an asterisk in the figures and tables. Other statistical analyses were also used: the Mann–Whitney test and Tukey’s test.

## 3. Results

To clarify the impact efficiency of melittin on erythrocyte and mitochondrial membranes at melittin concentrations that are sub-lytic and pre-pore forming, we present Table 1 containing the experimental assays independent of melittin treatment dose.

### 3.1. Colloid Osmotic Hemolysis

Colloid osmotic hemolysis of erythrocytes was determined spectrophotometrically, by studying the osmotic hemolysis and rate of hemolysis of cells in the presence or absence of melittin in an environment with different salt concentrations. The significance of surfactant-induced hemolysis has been demonstrated by Karabaliev et al. [71] to monitor changes in the morphology and oxygenation state of human erythrocytes. The methodology for determining osmotic hemolysis was described in detail in the articles [24,48,72].

**Figure 1:**
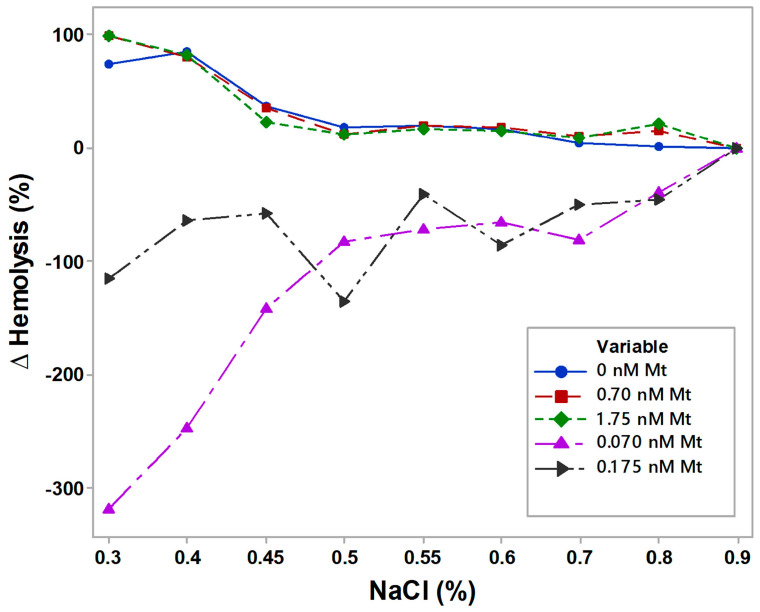
Hemolysis (hemoglobin release) of erythrocytes in the presence of different doses of melittin (Mt) at λ = 576 nm. Suspending media contained fixed concentrations of salt solutions. SD < 10%.

The osmotic curve of erythrocytes (Hct = 15%) without melittin in saline solutions indicated that the parameter hemolysis 50% was located at 4.2% NaCl (Figure 1). Melittin at a concentration of 0.70 nM led to 50% hemolysis at 4.18% NaCl, and a dose of 1.75 nM caused the value of 50% hemolysis to 4.15% NaCl. Thus, the parameter 50% hemolysis was slightly shifted to the left of the control hemolytic curve of erythrocytes in norm (4.2% NaCl) without significant changes in Hb-released under Mt-treatment.

### 3.2. Kinetics of Erythrocyte Hemolysis Under Melittin Treatment

**Figure 2:**
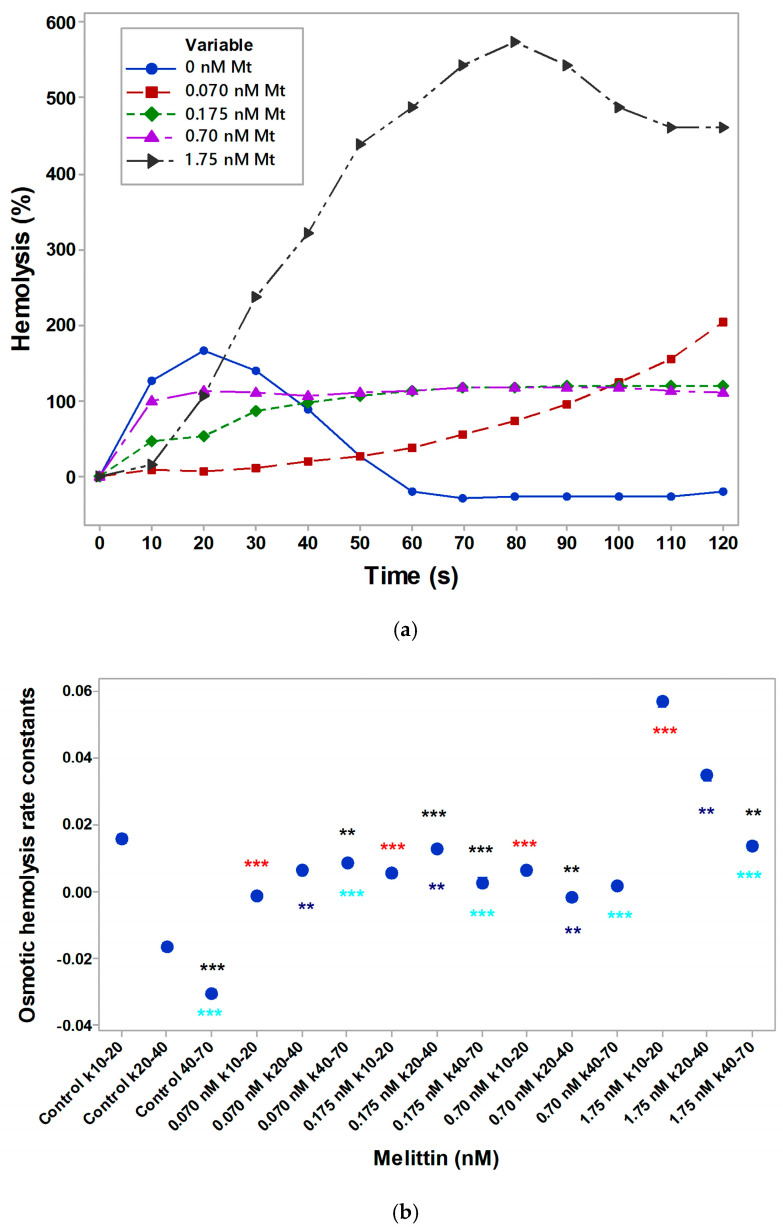
(**a**) Kinetics of erythrocyte hemolysis with different concentrations of melittin (Mt). Erythrocytes were suspended in 0.5% NaCl solution. (**b**) Mean values of erythrocyte osmotic hemolysis rate constant under different concentrations of melittin. SD < 10%. Each value represents the mean ± SD of three independent preparations (n = 3), averaged over three repetitions. Comparison of the effects of erythrocyte hemolysis at 0.5% NaCl versus the means of appropriate doses of melittin, the statistics obtained were as follows: rate constants k_10–20_ are colored blue, k_20–40_ are shown in red, k_40–70_ are colored cyan.

To account for the rate of osmotic hemolysis, monitoring was also required at 0.5% NaCl. Most distinct results for the rate of hemolysis were obtained using hypotonic medium with 0.5% NaCl. The kinetics of hemolysis correlated with erythrocyte osmotic fragility, which is thermodynamically governed by water influx into osmotically unbalanced cells [73]. Water influx typically leads to membrane destabilization and hemolysis. Thus, osmotic fragility is determined by the ratio between the initial cell volume and the critical volume at which membrane rupture occurs. Hemolysis kinetic curves of erythrocytes, suspended in 0.5% NaCl, with or without Mt, are shown in Figure 2a,b. No release of hemoglobin from erythrocytes was detected in a saline solution of 0.7–0.9% NaCl. In a hypotonic medium, a significant change in the hemolysis (at λ = 690 nm) of erythrocytes suspended in saline medium was observed in the direction of its reduction in the presence of doses of 0.070 nM (*p* = 0.0005) and 0.175 nM (*p* = 0.0041) Mt, respectively. Similar changes during hemolysis of the controls (without melittin) and the presence of 0.70 nM and 1.75 nM Mt indicated the absence of hemoglobin release from erythrocytes placed in saline solutions from 0.3% to 0.9% NaCl.

The elevated rate constant value of erythrocyte samples treated by 1.75 nM Mt correlated with the increased osmotic fragility (k_10–20_). There was a decrease in rate constant values (k_10–20_) of cells after treatment with doses of 0.070, 0.175 and 0.70 nM Mt (Figure 2b). There was an enhancement of mean values of erythrocyte osmotic hemolysis rate constant between 20 s and 40 s (k_20–40_) after administration of 1.75 nM Mt. The smaller effect of osmotic hemolysis rate constant between 40 s and 70 s (k_40–70_) was detected at 1.75 nM Mt concentration of treatment. The presence of 1.75 nM Mt in erythrocytes suspended in a saline solution of 0.5% NaCl showed significant changes (*p* <0.05) in the kinetics of hemolysis relative to the value of the Mt-less control. Erythrocytes treated with 1.75 nM Mt differed from these cells in the presence of 0.070 nM Mt, 0.175 nM Mt and 0.70 nM Mt at 0.5% NaCl (*p <* 0.05). The Mann–Whitney test, tracking the development of hemolysis of erythrocytes in a saline solution of 0.5% NaCl, shows its significant decrease in the presence of 0.175 nM Mt (*p* = 0.0038).The rate constants without and in the presence of fixed concentrations of melittin (0.070 nM; 0.175 nM; 0.70 nM; 1.75 nM), placed in a salt medium, (0.5% NaCl) were significantly different (*p <* 0.001) in the direction of increasing value (one-way ANOVA, SNK test). The rate constants k_20–40_ without and with fixed doses of 0.070 nM, 0.175 nM, 0.70 nM and 1.75 nM melittin in a saline solution of 0.5% NaCl showed a similar trend of increasing osmotic hemolysis (*p* = 0.009 according to one-way Anova tests) and tracked changes depending on the concentration of impact with melittin. The direction of the increase in the rate constant k_40–70_ in the presence of the above concentrations of melittin was also similar (*p <* 0.001, SNK test, one-way Anova). Tracking the changes in the rate constants (k_10–20_; k_20–40_; k_40–70_) showed the following features: k_10–20_ differed significantly from k_40–70_ (*p* = 0.004, Tukey test, *p <* 0.050). Changes in the rate constants k_10–20_ vs. k_20–40_ and k_20–40_ vs. k_40–70_ had no significant differences. The presence of 0.070 nM Mt in the erythrocyte sample at 0.5% NaCl led to an increase in the rate constant k_40–70_ compared to k_10–20_ (*p* = 0.004, Tukey test, *p <* 0.050). Comparing the rate constant values, k_10–20_ vs. k_20–40_ (*p* = 0.004, Tukey test, *p <* 0.050) and k_20–40_ vs. k_40–70_ were not significantly different in the presence of 0.070 nM Mt. The rate constants (k_10–20_; k_20–40_; k_40–70_) of erythrocytes in the presence of 0.175 nM melittin changed significantly (*p <* 0.001, SNK test at *p <* 0.05) when statistically comparing the values with each other. Comparing the rate constants (k_10–20_ vs. k_20–40_) in the presence of a dose of 0.70 nM Mt decreased (*p* = 0.004, Tukey test, *p <* 0.050), and did not differ when comparing k_20–40_ vs. k_40–70_ and k_10–20_ vs. k_40–70_. A dose of 1.75 nM Mt was found to decrease k_10–20_ compared to k_40–70_ (*p* = 0.004, Tukey test, *p <* 0.050). No differences were observed when statistically comparing the rate constants k_10–20_ vs. k_20–40_ and k_20–40_ versus k_40–70_.

### 3.3. Acid-Induced Hemolysis

The control kinetics of erythrocytes without melittin in the acidic medium (0.8% NaCl and 0.004 N HCl) was characterized by a peak of 360 s and a gradual decrease from 390 s to 510 s. The presence of melittin at all doses caused the acid curves to shift to the left, which meant a decrease in the acid fragility of erythrocytes in the presence of melittin and affected the older erythrocytes. Erythrocytes in the presence of 0.070 nM had a peak of their acid curve at 270 s, after which it dropped gradually to 450 s. The erythrocyte suspension under the influence of 0.175 nM showed a similar change in the acid curve with a peak at 270 s and a decrease from 300 s to 420 s. A dose of 0.70 nM of melittin led to an increase in the change in acid hemolysis by 35% at 360 s, while a concentration of 1.75 nM of melittin marked a peak of 35% of acid hemolysis at 300 s. The exponential drop in acid hemolysis ended at 450 s at a dose of 0.70 nM and at 390 s at 1.75 nM Mt in the erythrocyte suspension.

**Figure 3:**
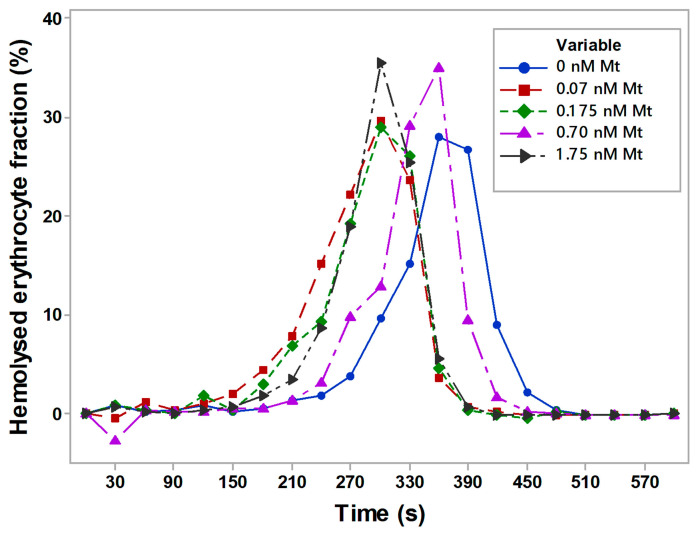
Acid-induced fragility of erythrocytes under different concentrations of melittin (Mt) treatment. Suspending medium consisted of 0.8% NaCl and 0.004 N HCl; control sample contained saline (0% acid-hemolysis changes). Each value of the erythrogram represents the mean ± SD of three independent preparations (n = 3).

Human erythrocytes were characterized by a peak, characterized by a fragility of the younger and more fragile erythrocyte membranes. Melittin led to a decrease in acid hemolysis of the older and less fragile red blood cells compared to the control (without melittin in suspending medium). The peak was slightly shifted to the left side of the erythrograms in the presence of all the doses of melittin. The peak shifted to the left side of the erythrograms was characterized by the strong influence of melittin (0.070 nM and 1.75 nM), where a strong decrease in the fragility of the older erythrocytes by 7.4-fold compared to the control (untreated ones) is shown (Figure 3).

Melittin influenced younger erythrocytes, which was clearly expressed at the lower concentrations of 0.070 nM and 0.175 nM doses on acid fragility of cells. Higher doses of 0.070 nM and 0.175 nM Mt increased the variation in acid hemolysis due to the stronger effect of melittin on erythrocyte hemolysis in the presence of hydrochloric acid as a hemolytic in the sample.

### 3.4. Effect of Melittin on Electrokinetic Properties of Erythrocytes

In experimental practice, there was no distinction made in the electrophoretic behavior of erythrocytes of different shapes (discocytes and echinocytes). A significant decrease in the surface electrical charge of red blood cells has been established during the storage of bank’s human blood [74,75]. Changes in the surface electrical charge simultaneously provided information about the pathological changes in the erythrocyte membrane [24,76,77]. This study presented data on electrophoretic mobility, zeta potential, and surface electrical charge of functionally active erythrocytes from human blood at the initial biophysical parameters of blood storage, such as at the end (21st day), and data on changes in the parameters after the end of the use of the blood bank (after 30 days of blood storage). At the same time, clinical data showed that the change in the normal ratio of erythrocytes of different shapes was associated with pathological changes, reflecting the overall health behavior of the individual [78,79]. In the first days of blood storage (days 1 to 5), a nearly constant value of electrophoretic mobility was recorded. Therefore, the cells we studied during this period were referred to as control erythrocytes. It was established that between days 5 and 10, a decrease in the measured value was recorded. After the tenth day until the end of the storage period (21 days), a slight and unreliable decrease in electrophoretic mobility was observed. These changes in the surface electrical charge were similar to those observed [74]. The observed dependence could be related to both the process of aging of the blood cells in vivo and to the biochemical changes in the environment during prolonged incubation of the blood cells. In this way, the studies conducted on the influence of bee venom melittin on erythrocytes during in vivo aging allowed the possibility for a deeper understanding of the action of melittin on cells. This enhanced the informational capabilities of the method of microelectrophoresis regarding the dynamics of the surface electrical charge as a result of changes in the structure and functional activity of the cells. Measurement of the electrophoretic mobility of erythrocytes or mitochondria in the presence of exogenously added melittin would allow us to detect changes in cell or vesicle shape as well as in their electrokinetic potential. The binding of lectins to the biological membrane in the presence of FITC-labeled CoA indicated changes in the structure of the membrane complexes under melittin were applied in order to determine the morphological changes. Comparison of the effect of different doses of melittin on the surface electrical properties of erythrocyte or mitochondrial membranes could clarify their mechanism of action on biological membranes. Erythrocytes and mitochondria with or without different concentrations of melittin were immediately measured for determination of its electrokinetic parameters. The upper membranes carried a negative surface electrical charge when suspended in the appropriate buffer solutions during microelectrophoretic studies and surface characteristics calculations. Experiments were conducted during in vivo aging of erythrocytes (functionally active cells) or in vitro aging of non-intact mitochondria (functionally inactive or uncoupled mitochondria) after isolation.

The electrokinetic study of erythrocytes was isolated from in vivo aging red blood cells named as modified erythrocytes. In this way, we fixed two measurement states of human erythrocytes as functionally active (erythrocytes in norm) and those that possess unaltered electrokinetic properties during in vivo aging of red blood cells (erythrocytes) during storage noticed as (in vivo aging of erythrocytes).

**Figure 4:**
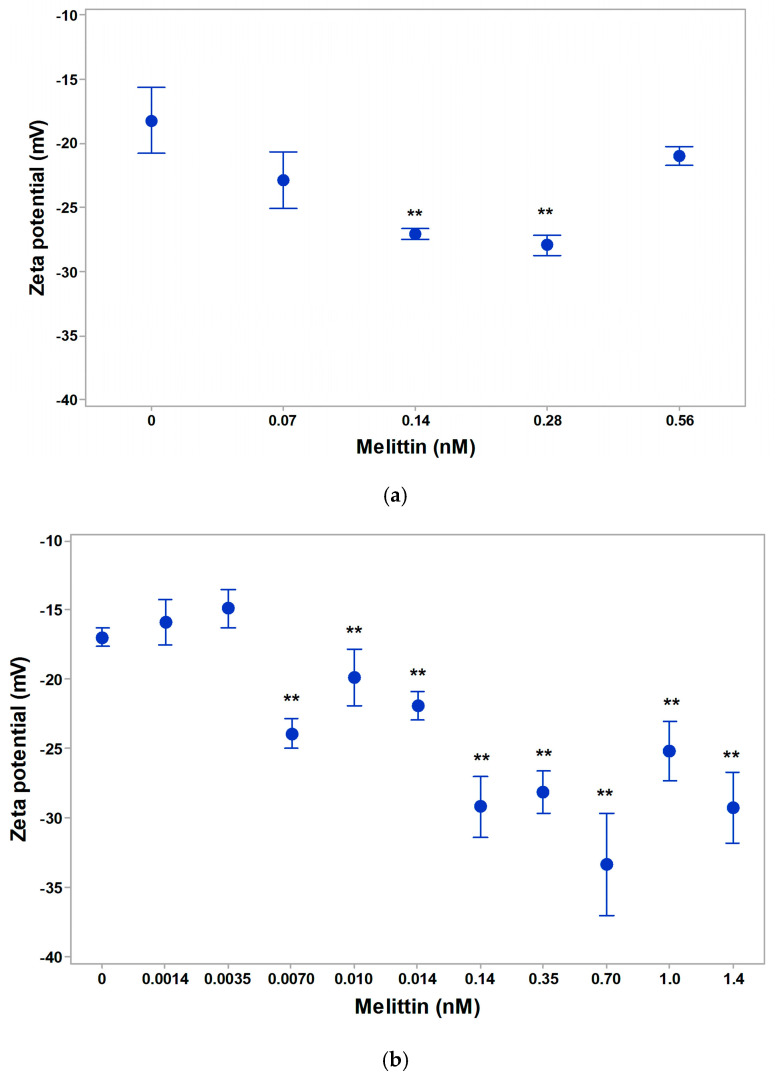

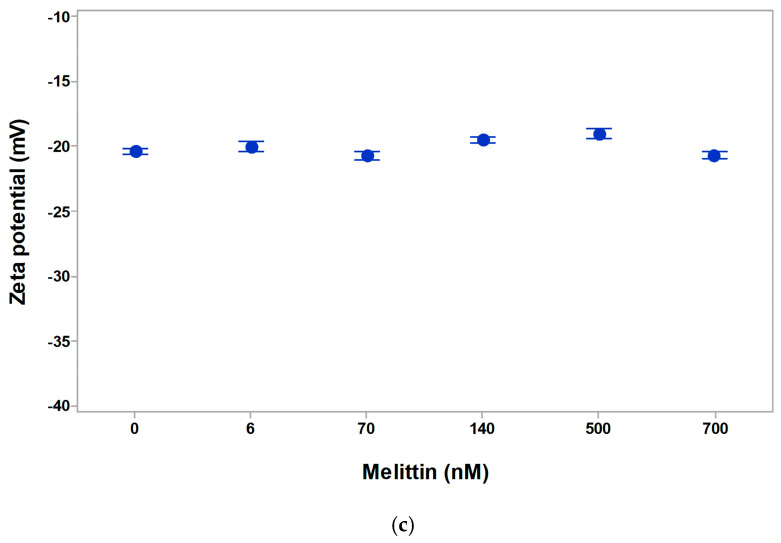
Effect of melittin on zeta potential of native erythrocytes (**a**), in vivo aging of erythrocyte membranes at 21st day of blood storage (**b**), and after data of blood storage (**c**). The suspending medium contained phosphate-buffered saline (PBS: 10.1 mM Na_2_HPO_4_, 1.8 mM KH_2_PO_4_, pH 7.4) at 25 °C. Each value represents the mean ± SD of three independent preparations (n = 3); each of them averaged over three repetitions (**, *p* < 0.01).

A strong effect of increase in zeta (electrokinetic) potential of 0.14–0.56 nM melittin in isotonic suspending medium of erythrocytes was established (Figure 4a). Low concentrations of melittin on the erythrocyte membrane of 0.0014 nM and 0.0035 nM led to a decrease in the negative zeta potential by 1.1 mV and 2.1 mV, respectively. This effect was due to the electrostatic binding of melittin at low concentrations of action on the surface of the erythrocyte membrane (Figure 4b). With increasing doses of melittin treatment, there was a gradual and strong increase in the negative zeta potential of erythrocytes by 6.9 mV (at 0.0070 nM) and by 12.2 mV (at 0.14 nM). The electrokinetic potential of erythrocytes increased by 11.3 mV when treated with 0.35 nM of melittin. The effect of melittin was most pronounced at doses of 0.70 nM, where the zeta potential of cells reached the value of –33.5 mV and increased by about 16.7 mV compared to the control value of erythrocytes, without melittin in the suspending medium. Melittin had a strong effect on cells with different functional states. Melittin could affect the increase in negative surface charge density due to an increase in the electrophoretic mobility of erythrocytes in the concentration range of (0.0014–1.40 nM). A non-significant decrease in the negative zeta potential (by 1.4 mV) of erythrocytes was found during their in vivo aging.

### 3.5. Effect of Melittin on Electrokinetic Properties of Mitochondria

Melittin was found to cause a decrease in the negative zeta potential of intact mitochondria when treated with nanomolar concentrations. The electrophoretic mobility of mitochondria obtained immediately after their isolation was determined, which characterized them as functionally active. We had labeled these mitochondria as intact. Isolated intact mitochondria were characterized by very low hydrolase activity. Added to intact mitochondria, 2.4-dinitrophenol (DNP) caused a strong stimulation of ATPase activity (7–10-fold), without this being associated with membrane destruction.

In our experiments, ATPase activity of isolated mitochondria was low during the registration for 180 s under control conditions. Addition of the uncoupler DNP at a final concentration of 50 µM strongly stimulated ATP hydrolysis, indicating a normal functional state of the mitochondria and low permeability of their inner membranes. Mitochondria isolated from rat liver maintained a high degree of coupling for more than 3 h after isolation and are normally used for biochemical and biophysical studies [80].

**Figure 5:**
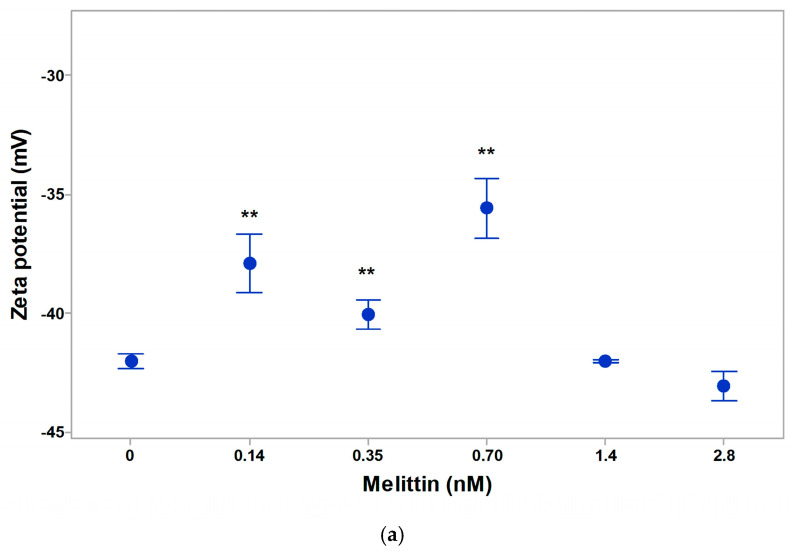

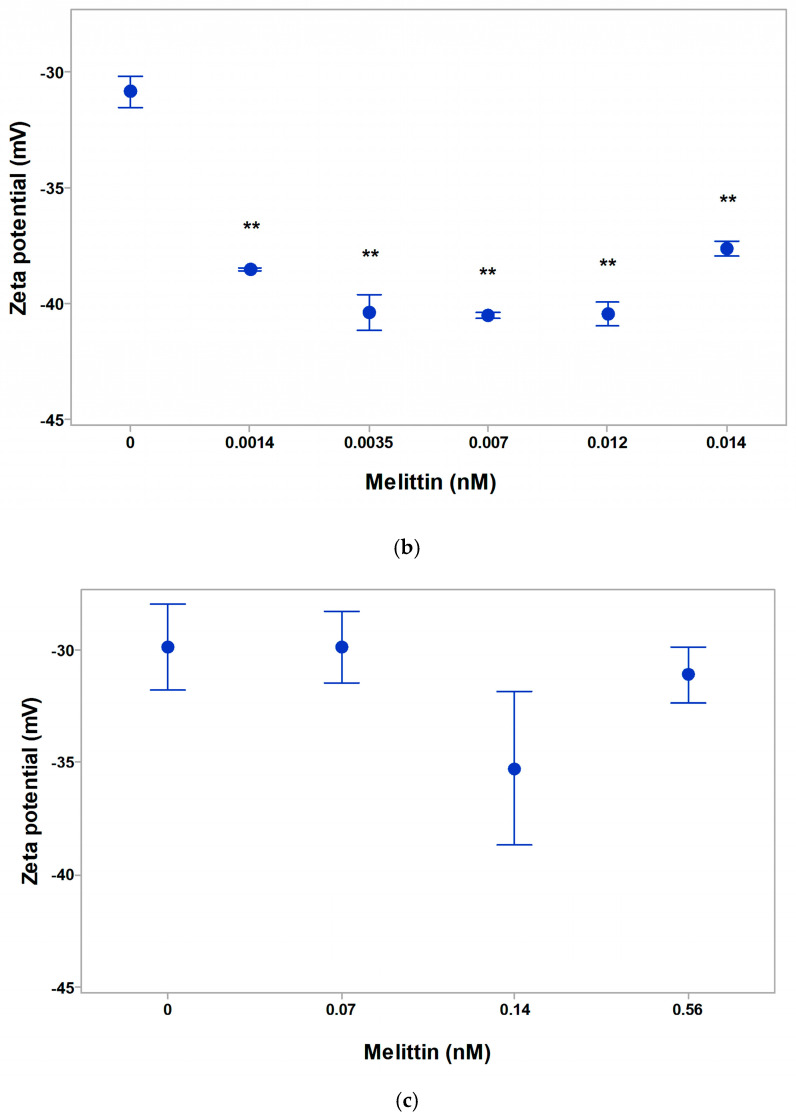
Effect of melittin on the zeta potential of rat liver intact mitochondria (**a**), non-intact mitochondria (**b**), 1 day after isolation or (**c**), 2 days after isolation. The suspending medium contained saline sorbitol buffer (SSB: 4.5% sorbitol, 14.50 mM NaCl, 0.6 mM NaCO_3_, pH 7.2 at 25 °C). Each value represents the mean ± SD of three independent preparations (n = 3); each of them averaged over three repetitions (** *p* < 0.01).

The zeta potential of intact mitochondria following melittin treatment is shown in Figure 5a. At physiological values of pH (7.2) and low ionic strength of the suspending media, melittin reduced the effect on the zeta potential of intact mitochondria. The effect could be due to the electrostatic anchorage of the peptide at the mitochondrial membrane. The zeta potential of intact mitochondria was most strongly reduced in the presence of 0.70 nM Mt in the suspending medium. The treatment of intact mitochondria with a dose of 2.8 nM Mt was characterized by a small part of the vesicles, with a zeta potential of –43.05 mV and many aggregates, which made the determination of their electrophoretic mobility more complicated. There were non-significant changes in zeta potential in mitochondrial suspensions under 2.8 nM Mt-treatment. The zeta potential of mitochondrial membranes at melittin doses of 1.4 and 2.8 nM are shown in Figure 5a. The data indicate that, at these low concentrations, melittin did not alter the zeta potential compared to the control, and no changes in conductivity of mitochondrial suspension were observed. 

In vitro mitochondrial aging, measured on the first and the second day after isolation of mitochondria is shown in Figure 5b,c. The zeta potential, electrophoretic mobility and surface electrical charge of non-intact mitochondria were characterized by a reduced value of electrokinetic potential, which confirmed the data of Navaro and Boveris [81] for dysfunctional mitochondria of aged mammalian tissues. This was reflected in the electrokinetic potential of the mitochondria on the first day after isolation, that is, more than 3 h post-isolation, a pronounced increase in the negative zeta potential of mitochondrial vesicles was observed. Non-intact mitochondria increased their electrokinetic potential by about 7–9 mV, which was due to an increase in the negative surface electrical charge of the outer mitochondrial membrane under the influence of melittin (0.0014–0.014 nM). That effect was also related to a strong reduction in the zeta potential of non-intact mitochondria (−30.91 mV) compared to that of intact mitochondria (−42.05 mV) by about 12 mV. Melittin can bind to the main components of the membranes and detect additional negatively charged groups exposed on the surface of the mitochondrial membrane, as no increase in lipid peroxidation products was observed in non-intact mitochondria had been registered. After two days of isolation, the electrokinetic parameters of mitochondria showed only non-significant changes (Figure 5c).

### 3.6. Membrane Transport of Erythrocytes in the Presence of Melittin

There was a rapid and concentration-dependent decrease in anion exchange and anion–proton co-transport. There was a reduction in proton uptake in the intracellular proton concentration upon melittin treatments (0.035–0.14 nM) as a function of time (s) calculated from the external pH due to the process of antiport of Cl^−^ and HCO_3_^−^ anions across the membrane compared to the control non-treated with melittin erythrocytes suspended in sucrose medium.

Melittin led to the inhibition of membrane transport across Band 3 of the erythrocyte suspension based on the comparison of the action of two agents at higher concentrations (ethanol and Ca^2+^ ions) considered to inhibit membrane transport. Ethanol could interact directly with membrane transport proteins, causing conformational changes that influenced their function. Ethanol inhibited outward Na^+^, K^+^ co-transport and the influx of LiCO_3_^–^ through the anion carrier (Band 3 protein) at a high concentration above 32 mM in in vitro studies [82]. The negative control of erythrocytes in the presence of 171 mM ethanol in suspending medium was presented to show the reversible changes in specific ion transport system. Calcium influenced erythrocyte membrane transport by activating channels and stimulating cell shape. At 1 mM CaCl_2_ exogenously added to the erythrocyte suspension in salt-free sucrose medium we expected that high intracellular calcium levels could lead the opening of Gardos channels, causing potassium loss, cell shrinkage, and dehydration as in the case of sickle cell anemia, observed by the literature data [83]. That was demonstration of the action of “electrostatic” second negative control, which showed the inhibition of membrane transport at the dose of calcium ions in erythrocyte suspension.

A positive control (oxytocin) was also used which direct effect on erythrocyte transport led to the release of calcium ions into the extracellular space and affected calcium transport in other cell types. There was insufficient data in the literature on the effect of oxytocin on the mechanism of direct and significant effect of oxytocin on specific transport of erythrocytes under normal physiological conditions. The positive control of 2.014 g/mol oxytocin represented an activation of proton transport across the erythrocyte membrane in physiological pH of 7.4 during time of membrane transport processes. All studies of membrane transport were conducted with the direct introduction of chemical agents, i.e., without prior incubation of erythrocytes with the specified substances. In this way we tracked the direct action of chemical agents on membrane transport through the erythrocyte membrane at room temperature of 25°C.

**Figure 6:**
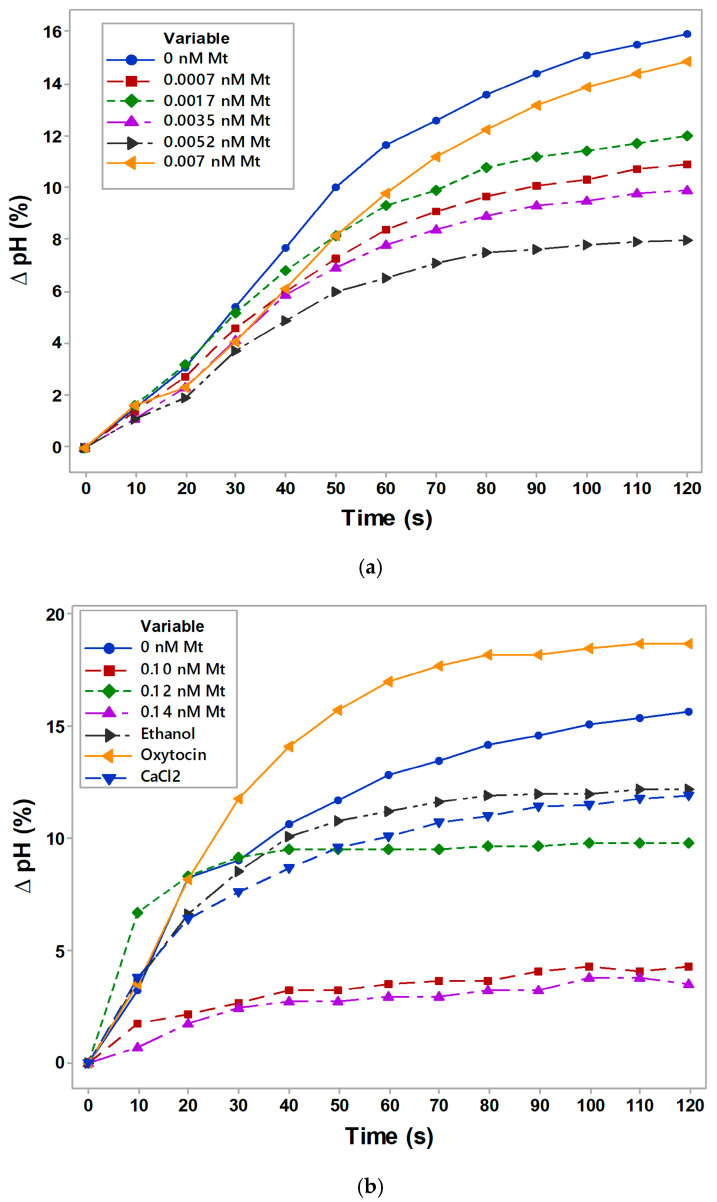

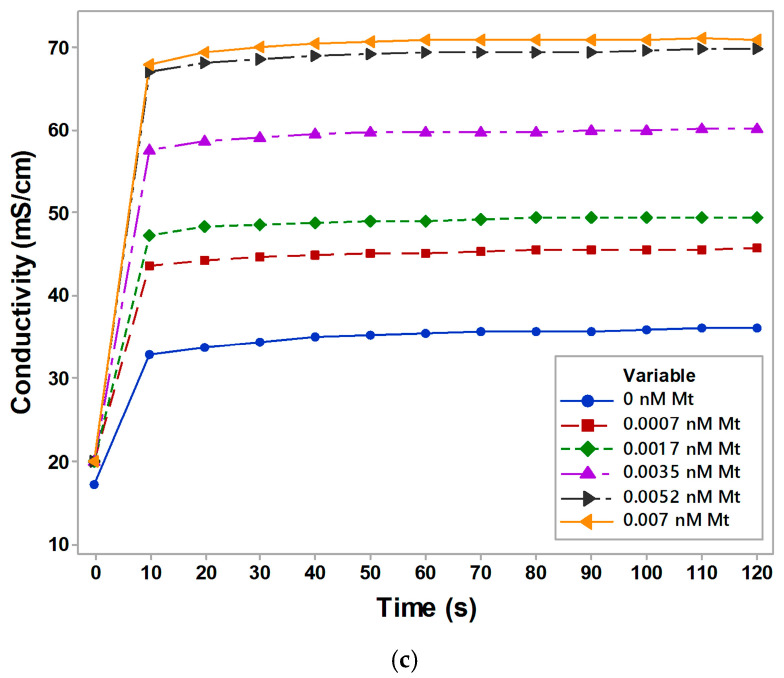
Kinetics of ΔpH changes (**a**,**b**) and conductivity (**c**) of erythrocyte suspension with or without melittin. Extracellular proton concentration (H^+^_ext_) as a function of time (s), calculated from the recorded ∆pH changes induced by the sudden jumps of the extracellular proton concentration of erythrocytes with or without different doses of melittin (Mt). Negative controls were applied in the presence of ethanol (171 mM) or 1 mM calcium chloride (CaCl_2_). A positive control was also prepared in the presence of oxytocin (2.014 g/mol). Mann–Whitney test for medians of control erythrocytes vs. that of 0.0052 nM Mt was determined (*p <* 0.05, (**a**)). All the medians of the conductivity of erythrocyte suspension (0.3 M sucrose, pH = 7.4 (NaOH)) were detected (*p <* 0.05; *p <* 0.01 by Mann–Whitney test for nonparametric analyses of the values). Membrane transport measurements were performed at room temperature of 25 °C in 0.3 M sucrose, pH 7.4 (NaOH). Each value represents the mean ± SD of three independent preparations (n = 3).

Significant differences were found in the membrane transport of erythrocytes in the presence of 0.0052 nM Mt in the suspension medium (*p <* 0.05) in time up to 240 s. Significant changes in ∆pH were observed without (*p* = 0.0014, Mann–Whitney test) and in the presence of 0.12 nM Mt (*p* = 0.0483), 0.14 nM Mt (*p* = 0.0006), as well as in the presence of positive control with oxytocin (*p <* 0.053, Figure 6b). Negative controls with ethanol and with CaCl_2_ did not show significant changes at *p <* 0.05 according to the Mann–Whitney test. Changes in membrane transport were characterized by strong changes in the conductivity of the erythrocyte suspension in the presence of all the concentrations of melittin (0.0007–0.007 nM Mt) in a medium containing salt-free sucrose, pH 7.4 (NaOH) (*p* = 0.0002, adjusted for ties), (Figure 6c).

Based on the results obtained, melittin at sub-lytic, pre-pore formation concentrations might induce conformational changes in the Band 3 protein as a result of altered proton transport.

### 3.7. Lipid Peroxidation of Erythrocytes and Mitochondria in the Presence of Melittin

**Figure 7:**
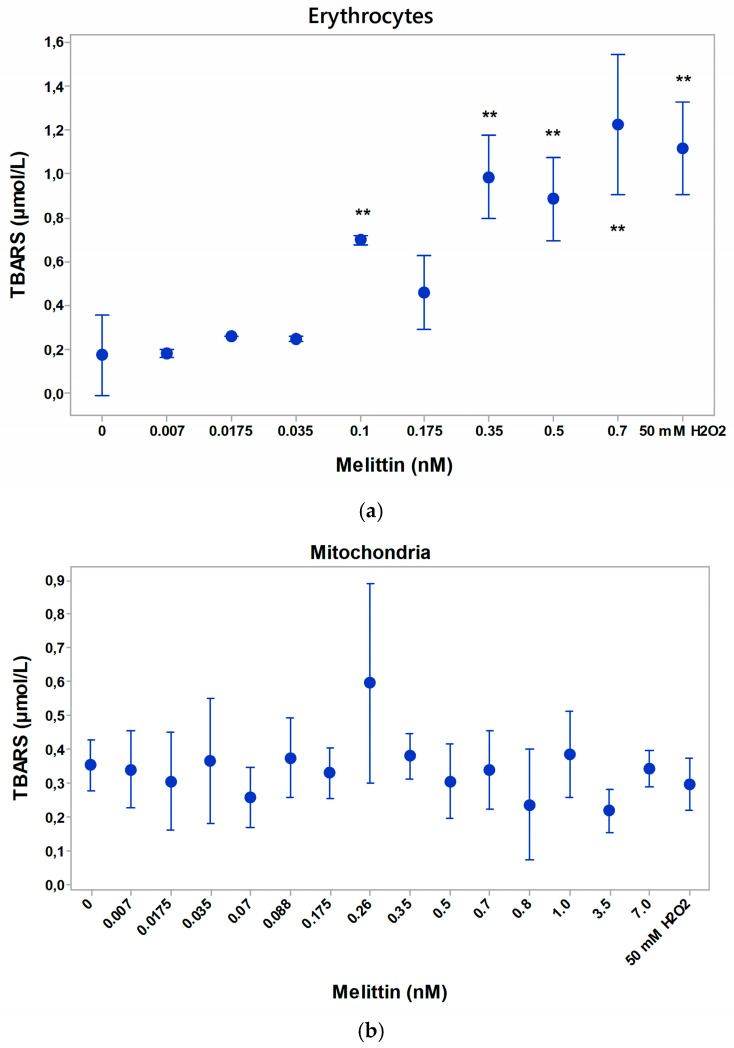
Lipid peroxidation of erythrocytes in norm under melittin treatment (**a**). TBARS of erythrocytes, suspended in PBS, pH 7.4, 2 mM NaN_3_. Each value of the biophysical measurements was the mean ± SD of the three independent preparations (n = 3), with each of them averaged over three repetitions at ** *p <* 0.01. The second positive control with 50 mM H_2_O_2_ in erythrocyte suspending medium was used. Lipid peroxidation of rat liver mitochondria under different doses of melittin treatment (**b**). TBARS of mitochondria, suspended in 50 mM Tris-HCl, 150 mM KCl at pH 7.4 at room temperature of 25 °C. A second control of mitochondria was presented after treatment with 50 mM hydrogen peroxide (H_2_O_2_). Each value represents the mean ± SD of three independent preparations with more than five repetitions each.

An increased amount of TBARS products from the peroxidation of erythrocyte lipids in the presence of melittin (0.035–0.14 nM) in the suspending medium was determined (Figure 7). The increased negative zeta potential of erythrocytes after treatment with melittin was associated with the formation of an increased level of lipid peroxidation products of the membrane.

No change in mitochondrial lipid peroxidation was observed before and after treatment with a wide range of melittin concentrations (Figure 7). The observed effects of melittin (0.007–0.7 nM) on the electrokinetic parameters of mitochondria were not associated with a significant change in the formation of lipid peroxidation products compared to those of untreated membranes.

Based on the literature data on the influence of phospholipase A2 at low-exposure concentrations, we could report that the increased lipid peroxidation in the presence of nanomolar concentrations was accompanied by the change in the electrokinetic potential based on the change in the surface electrical charge. The formation of radicals generated from the lipid phase of the membrane based on the accumulation of MDA content gave information about additional polarization of the erythrocyte membrane.

### 3.8. Fluorescence Microscopy of Erythrocytes and Mitochondria Under Melittin Treatment

**Figure 8:**
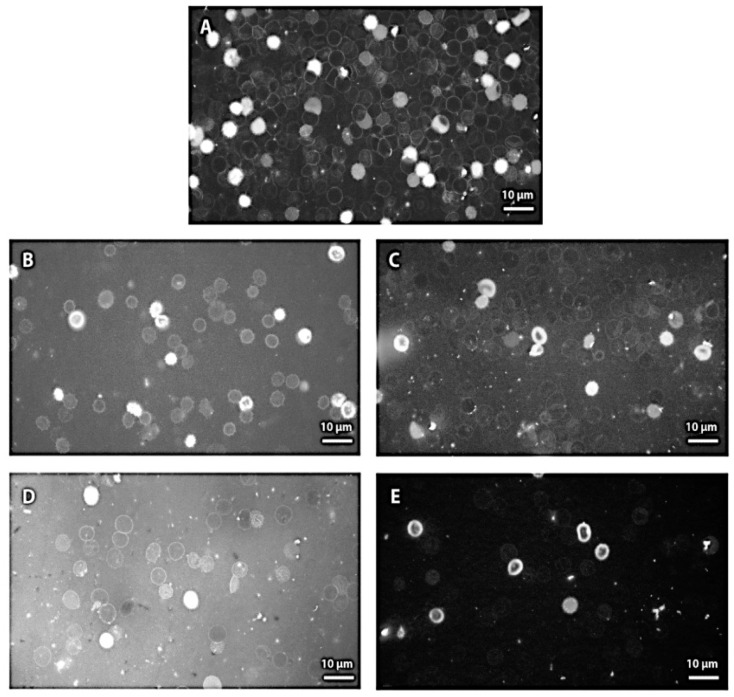
Fluorescence microscopy images of human erythrocytes without (control, (**A**)) and at different Mt concentrations: (0.070 nM Mt, (**B**)); (0.175 nM Mt, (**C**)); (0.7 nM Mt, (**D**)); (1.75 nM Mt, (**E**)). Scale bars, 10 µm.

Fluorescence microscopy images showed the observed morphological changes in human erythrocytes induced by melittin peptides at concentrations of (0.070; 0.175; 0.70; 1.75 nM) (Figure 8). The transformation of crenated form was observed after addition of 0.070 and 0.175 nM Mt (Figure 8A,B). Echinocytes, swollen erythrocytes with a rounded cell shape were observed, and no difference in erythrocyte shapes was observed at the studied doses of 0.070 and 0.175 nM Mt in erythrocyte suspension.

The transformation of erythrocyte shape into a spiculate form at a lytic concentration of melittin was established by Tostesson and co-authors [84]. Melittin was able to deform the erythrocyte into crenature which made the cell unstable with release of membrane fragments and the enhancement of cell’s permeability [14].

The higher melittin concentrations of 0.70 and 1.75 nM caused the transformation of erythrocytes into a round shape with protrusions, erythrocyte shadows with a small and large radius of the cell. Erythrocytes with good staining on the surface of the membrane were also observed, which looked like cells with a well-defined halo around them. Fewer erythrocytes were also found compared to the control, untreated cells. A dose of 1.75 nM Mt produced a shape change that was shifted to one end of the cell and prominent protrusions to a greater extent compared to the shape of erythrocytes treated with 0.70 nM Mt (Figure 8C). The cells were more swollen, with the protrusions affecting a larger portion of the erythrocytes, and the concave portion shifted toward one pole of the cell. In some of the cells after treatment by 1.75 nM melittin, the invagination in shape was observed resulting from undulating movements of cellular components. Various transformations were seen on the surface of erythrocytes after treatment with 1.75 nM Mt (Figure 8D).

Fluorescence microscopy studies showing changes in erythrocyte shape from normal discoid to echinocyte supported the hypothesis of the group of Katsu and co-workers [14]. According to this hypothesis, a change in membrane permeability was found just prior to the release of membrane components in the presence of 0.05 µM melittin [14]. Thus, peptide molecules of melittin accumulated on the outer membrane of the erythrocyte caused the formation of spicules and protrusions based on conformational changes in the protein of Band 3 without the presence of hemolysis at physiological pH values.

**Figure 9:**
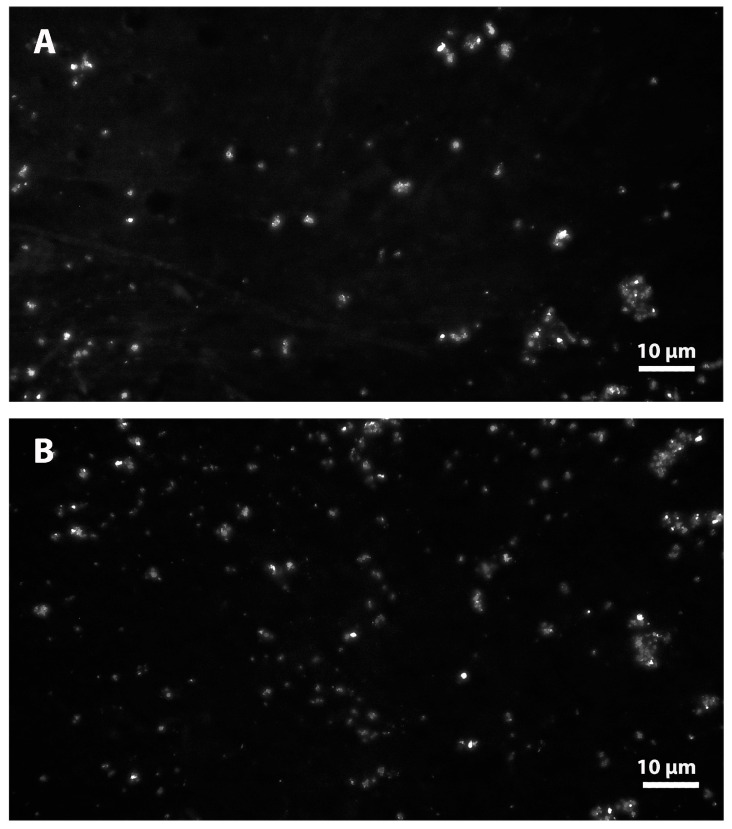
Isolated rat liver mitochondria after FITC-concanavalin A labeling. Representative fluorescent images of mitochondria without (control, (**A**)) and under 0.1 nM melittin (**B**). Scale bars, 10 µm.

Fluorescence microscopy of mitochondria is one of the most valuable tools for investigating mitochondrial bioenergetics and determination of mitochondrial membrane potential [85]. Mitochondrial vesicles were evenly distributed in the fluorescence microscopy image of mitochondria without melittin in the suspending medium (Figure 9A). The decrease in fluorescence intensity of mitochondria after treatment with 0.1 nM Mt was accompanied by clustering of mitochondrial complexes (Figure 9B). These compacted mitochondrial clusters with halos from the binding of FITC—CoA to specific receptors on the outer surface of the membrane were related to the ability of melittin to assemble mitochondrial vesicles based on hydrophobic interactions. A slight enhancement of the light scattering of mitochondria suspended in low-ionic-strength medium in the presence of melittin was found as described in Doltchinkova and co-workers [86]. In the present study, we hypothesized that weak aggregation of mitochondrial vesicles could occur in a low-ionic-strength environment in the presence of sub-lytic concentrations of melittin.

## 4. Discussion

The electrical charges at the cell membrane surface play a crucial role in determining vascular wall permeability, the transport of ions and macromolecules, and interactions involved in intermolecular recognition processes. The initial interaction between melittin and the erythrocyte cells is primarily electrostatic, mediated by the basic C-terminus, with additional stabilization at the membrane plane, where the peptide adopts a low α-helical conformation [87,88]. The density of the surface electrical charge in membranes and subcellular organelles is a key factor governing their interaction with the extracellular environment as well as adhesion and aggregation processes. Electrostatic forces influence the orientation of membrane proteins, while asymmetry in surface charge arising from the uneven distribution of ions at the bilayer affects the proper positioning of peptides within the membrane. Transmembrane electric fields further modulate peptide conformation. Consequently, alterations in surface electrical properties, resulting from the binding of ions or melittin molecules to the membrane during aging, may induce changes in molecular organization and membrane function.

The study and analysis of the electrokinetic properties of erythrocyte and mitochondrial membranes, as well as the influence of melittin on these properties, were important for optimizing the conditions of membrane–melittin interactions. These interactions involve melittin’s attachment to or repulsion from the membrane surface, as well as aggregation within the cells. Melittin penetrates between lipid molecules, thereby expanding the membrane surface area. The study and analysis of the electrokinetic properties of erythrocyte and mitochondrial membranes were important for optimizing the conditions of membrane-melittin interactions. These interactions involve melittin’s attachment to or repulsion from the membrane surface. Melittin penetrates between lipid molecules thereby expanding the membrane surface area [32,89,90]. Treatment of human erythrocyte membranes with melittin in an isotonic ionic medium resulted in a pronounced increase in their negative electrokinetic potential, reflecting an enhancement of the negative surface charge.

Based on data showing that melittin segregates into hydrophobic and hydrophilic amino acid residues during its interaction with membranes [14,91], an increase in the conductivity of erythrocyte suspensions due to the release of membrane fragments was observed. Furthermore, drawing on literature data regarding the effect of phospholipase A2 at low melittin concentrations, we suggest that the observed increase in lipid peroxidation in the presence of nanomolar concentrations of melittin is accompanied by an increase in negative zeta potential.

Following melittin exposure, intact mitochondria exhibited a pronounced decrease in electrophoretic mobility, corresponding, to a reduction in the negative zeta potential compared to untreated controls. Accumulation of the peptide on the membrane surface altered electrokinetic stability, potentially increasing ion permeability. The effects of melittin on the mitochondrial membrane were more pronounced in a lower-ionic-strength medium. Previous studies have reported that melittin affects ATPase activity depending on its concentration in the reaction medium [86]. As melittin concentration increased, a corresponding rise in the total H^+^concentration in the solution was observed, reflecting stimulation of ATP hydrolysis, and this increased ATPase activity. In the presence of high ATP concentrations, the enzyme facilitates ATP degradation due to easier access to the inner mitochondrial membrane. It is well-known that, under conditions of excess ATP, ATP synthase can reverse its activity and function as an ATP hydrolase.

Melittin concentrations in the range of 0.04–8 µM have been reported to correspond to levels responsible for its membrane-disrupting and antimicrobial activities [39]. At higher concentrations, such as 10 µM, significant structural alterations were observed in DMPG and DMPS membranes [92]. In contrast, the presence of negatively charged lipids and proteins in mitochondrial membranes hinders the lytic activity of melittin.

Electrokinetic analysis provided valuable insights into how biological membranes respond to melittin exposure. By demonstrating that melittin alters membrane surface charge in parallel with aging-related changes in erythrocytes and mitochondria, our findings contribute to a more comprehensive understanding of the molecular mechanisms underlying its cellular effects. These results further highlight the relevance of melittin-induced electrostatic and oxidative membrane modifications to inflammatory responses and redox imbalance [93]. The presented results on the sub-lytic concentration-dependent relationship between surface charge and lipid peroxidation may help clarify the mechanisms linking melittin’s biophysical actions to its pathological and therapeutic potential.

## 5. Conclusions

We reported the divergent effects of sub-lytic, pre-pore-forming concentrations of melittin on surface properties of aging-associated erythrocytes and mitochondria. Changes in the electrostatic free energy of melittin-treated erythrocytes and mitochondria were quantified. Melittin induced notable alterations in erythrocyte morphology, attributable both to structural remodeling of the cells and to the attraction of subcellular elements toward the thermodynamic state characteristic of aggregated mitochondria. Moreover, melittin exposure increased the malondialdehyde content in erythrocyte membranes, whereas mitochondrial MDA levels remained unaffected. An increased conductivity of cell suspension was observed, reflecting reduction in proton transport across erythrocyte membranes. Overall, the electrokinetic characteristics revealed here provide insight into cellular responses to melittin and contribute to understanding the mechanisms underlying its pathological and biomedical activities.

## Figures and Tables

**Table 1:** Experimental assays for biophysical studying human erythrocytes and rat liver mitochondria in the presence of sub-lytic, pre-pore concentrations of bee venom melittin from *Apis mellifera*. IM, intact mitochondria; NIM, non-intact mitochondria.

**ERYTHROCYTES**										
**Experimental Assays**	**∆Hemolysis (%)**	**Osmotic Fragility & Rate** **Constants at 0.5% NaCl**	**Acid-Induced Hemolysis**	**Zeta** **Potential—9 d of storage**	**Zeta** **Potential—21 d of Storage**	**Zeta** **Potential After the Date of Blood** **Storage**	**Membrane Transport** **Δ pH (%)**	**Conductivity**	**Lipid** **Peroxidation**	**Fluorescence Microscopy**
Melittin, nM	0.07–1.75	0.07–1.75	0.07–1.75	0.07–0.56	0.0014–1.4	6–700	0.0007–0.14	0.0007–0.007	0.007–0.7	0.07–1.75
**MITOCHONDRIA**										
**Experimental Assays**				**Zeta** **Potential—Fresh IM**	**Zeta** **Potential—NIM-1d**	**Zeta** **Potential—NIM-2d**			**Lipid** **Peroxidation**	**Fluorescence Microscopy**
Melittin, nM				0.14–2.80	0.0014–0.014	0.07–0.56			0.007–7.0	0.1

## Data Availability

The original contributions presented in the study are included in the article, further inquiries can be directed to the corresponding authors.

## Abbreviations

The following abbreviations are used in this manuscript:

CoA	concanavalin A
DDL	diffuse double layer
EPM	electrophoretic mobility
FITC	fluorescein isothiocyanate
G^s^_el_	electrostatic free energy
Hb	hemoglobin
Hct	hematocrit
MDA	malondialdehyde
Mt	melittin
PBS	phosphate-buffered saline
PI	propidium iodide
SD	standard deviation
SSB	saline sorbitol buffer
TBA	2-thiobarbituric acid
TBARS	thiobarbituric acid reactive substances
Tris-HCl	Trizmahydrochloride, Tris [hydroxymethyl] aminomethan hydrochloride
σ	surface electrical charge
ζ	zeta (electrokinetic) potential

## References

[1] Hong J., Lu X., Deng Z., Xiao S., Yuan B., Yang K. (2019). How Melittin Inserts into Cell Membrane: Conformatonal Changes, Inter-Peptide Cooperation, and Disturbance on the Membrane. Molecules.

[2] Tamm L.K. (1991). Membrane insertion and lateral mobility of synthetic amphiphilic signal peptides in lipid model membranes. Biochim. Biophys. Acta.

[3] Hristova K., Dempsey C.E., White S.H. (2001). Structure, Location, and Lipid Perturbations of Melittin at the Membrane Interface. Biophys. J..

[4] Volovik M.V., Denieva Z.G., Kondrashov O.V., Akimov S.A., Batishchev O.V. (2025). Dialectics of Antimicrobial Peptides I: Common Mechanisms of Offensive and Protecting Roles of Peptides. Langmuir.

[5] Kondrashov O.V., Volovik M.V., Denieva Z.G., Gifer P.K., Galimzyanov T.R., Kuzmin P.I., Batishchev O.V., Akimov S.A. (2025). Dialectics of Antimicrobial Peptides II: Theoretical Models of Pore Formation and Membrane Protection. Langmuir.

[6] Raghuraman H., Chattopadhyay A. (2007). Melittin: A membrane-active peptide with diverse functions. Biosci. Rep..

[7] Takahashi T., Nomura F., Yokoyama Y., Tanaka-Takiguchi Y., Homma M., Takiguchi K. (2013). Multiple Membrane Interactions and Versatile Vesicle Deformations Elicited by Melittin. Toxins.

[8] Ulmschneider J.P., Ulmschneider M.B. (2024). Melittin can permeabilize membranes via large transient pores. Nat. Commun..

[9] Somase V., Desai S.A., Patel V.P., Patil V., Bhosale K. (2024). Antimicrobial Peptides: Potential Alternative to Antibiotics and Overcoming Limitations for Future Therapeutic Applications. Int. J. Pept. Res. Ther..

[10] Ceremuga M., Stela M., Janik E., Gorniak L., Synowiec E., Sliwinski T., Sitarek P., Saluk-Bijak J., Bijak M. (2020). Melittin -A Natural Peptide from Bee Venom Which Induces Apoptosis in Human Leukaemia Cells. Biomolecules.

[11] Stela M., Cichon N., Spławska A., Szyposzynska M., Bijak M. (2024). Therapeutic Potential and Mechanisms of Bee Venom Therapy: A Comprehensive Review of Apitoxin Applications and Safety Enhancement Strategies. Pharmaceuticals.

[12] Zhang S.F., Chen Z. (2017). Melittin exerts an antitumor effect on non-small lung cancer cells. Mol. Med. Rep..

[13] Bernheimer A.W., Rudy B. (1986). Interaction between membranes and cytolitic peptides. Biochim. Biophys. Acta.

[14] Katsu T., Kuroko M., Morikawa T., Sanchika K., Fujita Y., Yamamura H., Uda M. (1989). Mechanism of membrane damage induced by the amphipathic peptides gramicidin S and melittin. Biochim. Biophys. Acta.

[15] Beschiaschvili G., Baeuerle H.-D. (1991). Effective charge of melittin upon interaction with POPC vesicles. Biochim. Biophys. Acta.

[16] Xu Y., Hanlon P., Rael E.D., Gasanoff E.S. (2020). Bee Venom Melittin Modulates Phospholipase A2 Activity by Affecting Substrate Interface on the Surface of Phosphatidylcholine Membrane. Ann. Toxicol..

[17] Gasanoff E., Liu Y., Li F., Hanlon P., Garab G. (2021). Bee Venom Melittin Disintegrates the Respiration of Mitochondria in Healthy Cells and Lymphoblasts, and Induces the Formation in Model Inner Mitochondrial membranes. Int. J. Mol. Sci..

[18] Willyard C. (2017). The drug-resistant bacteria that pose the greatest health threats. Nature.

[19] Eisenburg S., Haimov E., Walpole G.F.W., Plumb J., Kozlov M.M., Grinstein S. (2021). Mapping the electrostatic properties of cellular membranes. Mol. Biol. Cell.

[20] Jarzynska K., Ciura K., Gao X.J., Mikolajczyk A., Gao X., Puzin T. (2025). Understanding the zeta potential of nanomaterials through predictive nanoinformatics. Nanotoday.

[21] Neu B., Sowemimo-Coker S.O., Meiselman H.J. (2003). Cell-Cell Affinity of Senescent Human Erythrocytes. Biophys. J..

[22] Wang M., Yang L., Wang X., Huang T., Jin Y., Ye L., Jiang Z., Chen J., Sun Z., Wang X. (2025). Transform in sialic acid charge inhibits cell migration depending on CD44-integrin crosstalk by remodeling cytoskeleton. Chem. Eng. J..

[23] Hughes M.P. (2024). The cellular zeta potential: Cell electrophysiology beyond the membranes. Integr. Biol..

[24] Doltchinkova V., Kitanova M., Nikolov R., Stoyanova-Ivanova A., Petkov O., Dikova Y., Vitkova V. (2023). Erythrocyte Membrane Biophysical Changes Mediated by Pooled Immunoglobulin G and Hematin: Electrokinetic and Lipid Peroxidation Studies. Membranes.

[25] Hincha D.K., Crowe J.H. (1996). The lytic activity of the bee venom peptide melittin is strongly reduced by the presence of negatively charged phospholipids or chloroplast galactolipids in the membranes of phosphatidylcholine large unilamellar vesicles. Biochim. Biophys. Acta.

[26] Irudayam S.J., Berkowitz M.L. (2012). Binding and reorientation of melittin in a POPC bilayer: Computer simulations. Biochim. Biophys. Acta.

[27] Hui S.W., Stewart C.M., Cherry R.J. (1990). Electron microscopic observation of the aggregation of membrane proteins in human erythrocytes by melittin. Biochim. Biophys. Acta.

[28] Hu K.-S., Dufton M.J., Morrison I.E.G., Cherry R.J. (1985). Protein rotational diffusion measurements on the interaction of bee venom melittin with bacteriorhodopsin in lipid vesicles. Biochim. Biophys. Acta.

[29] Shi H., Hu K.-S., Huang Y., Dong N.-X. (1993). Effect of melittin on photocycle and photopotential of purple membrane: Sites of interaction between bacteriorhodopsin and melittin. Photochem. Photobiol..

[30] Kamo N., Muratsugu M., Kurihara K., Kobatake Y. (1976). Change in surface charge density and membrane potential of intact mitochondria during energization. FEBS Lett..

[31] Woitczak L., Nałęcz M.J. (1979). Surface charge of biological membranes as a possible regulator of membrane-bound enzymes. Eur. J. Biochem..

[32] Mulkidjanian A.Y., Galperin M.Y., Makarova K.S., Wolf Y.I., Koonin E.V. (2008). Evolutionary primacy of sodium bioenergetics. Biol. Direct.

[33] Batenburg A.M., Hibbeln J.C., de Kruijff B. (1987). Lipid specific penetration of melittin into phospholipid model membranes. Biochim. Biophys. Acta.

[34] Doltchinkova V., Nikolov R., Shkodrova M., Stoyanova-Ivanova A., Petkov O., Vitkova V., Balaž A., Djordjiević G., Karamarković J., Lazarević N. (2022). Effect of Melittin on the Human Erythrocyte and Rat Liver Mitochondrial Membranes. Proceedings of the BPU 11 Congress The 11th International Conference of the Balkan Physical Union—“Biophysics and Medical Physics”, Belgrad, Serbia, 28 August—1 September 2022.

[35] Sae-Lee W., McCafferty C.L., Verbeke E.J., Havugimana P.C., Papoulas O., McWhite C.D., Houser J.R., Vanuytsel K., Murphy G.J., Drew K. (2022). The protein organization of a red blood cell. Cell Rep..

[36] Casares D., Escribá P.V., Rpsselló C.A. (2019). Membrane lipid composition: Effect on Membrane and Organelle Structure, Function and Compartmentalization and Therapeutic Avenues. Int. J. Mol. Sci..

[37] Ali O., Szabó A. (2023). Review of Eukaryote Cellular Membrane Lipid Composition, with Special Attention to Fatty Acids. Int. J. Mol. Sci..

[38] Krompers M., Jaki M., Götz S., Lembeck J., Kaul L., Holzer M., Heerklotz H. (2025). Asymmetric phase transitions in lipid bilayers: Coupling or bending?. Faraday Discuss..

[39] Schiaffarino O., Valdivieso González D., García-Pérez I.M., Penalva D.A., Almendro-Vedia V.G., Notale P., López-Montero I. (2022). Mitochondrial membrane models built from native lipid extracts: Interfacial and transport properties. Front. Mol. Biosci..

[40] Sabapathy T., Deplazes E., Mancera R.L. (2020). Revisiting the Interaction of Melittin with Phospholipid Bilayers: The Effects of Concentration and Ionic Strength. Int. J. Mol. Sci..

[41] Doltchinkova V., Georgieva K., Traytheva N., Slavov C., Mishev K. (2003). Melittin-induced changes in thylakoid membranes: Particle electrophoresis and light scattering study. Biophys. Chem..

[42] Dempsey C.E. (1990). The actions of melittin on membranes. Biochim. Biophys. Acta.

[43] Klocek G., Schulthess T., Shai Y., Seelig J. (2009). Thermodynamics of Melittin Binding to Lipid Bilayers. Aggregation and Pore Formation. Biochemistry.

[44] Stankowsli S. (1991). Surface charging by large multivalent molecules. Extending the standard Gouy-Chapman treatment. Biophys. J..

[45] Lee T.-H., Hall K., Aguilar M.-I. (2020). The Effect of Charge on Melittin-Induced Changes in Membrane Structure and Morphology. Aust. J. Chem..

[46] WMA Declaration of Helsinki – Ethical Principles for Medical Research Involving Human Participants. https://www.wma.net/policies-post/wma-declaration-of-helsinki/.

[47] Seaman G.V., Uhlenbruck G. (1962). Electrophoretic motility of erythrocytes after treatment with various enzymes and antisera. Klin. Wochenschr..

[48] Doltchinkova V., Stoylov S., Angelova P.R. (2021). Viper toxins affect membrane characteristics of human erythrocytes. Biophys. Chem..

[49] Kante A., Berrez J.M., Latruffe N., Latruffe N., Gaudemer Y., Vignais P., Azzi A. (1988). Synthesis and Maturation of D–β-Hydroxybutyrate Dehydrogenase (BDH) from Mitochondrial Inner Membrane. Dynamics of Membrane Proteins and Cellular Energetics.

[50] Waseem M., Tabassum H., Bhardwaj M., Parvez S. (2017). Ameliorative efficacy of quercetin against cisplatin-induced mitochondrial dysfunction: Study on isolated rat liver mitochondria. Mol. Med. Rep..

[51] Doltchinkova V., Vitkova V., Shkodrova M., Kitanova M., Lozanova V., Lozanova S., Ivanov A., Roumenin C. (2025). Mitochondrial alterations induced by *Macrovipera lebetina* venom. Eur. Biophys. J..

[52] (2010). Directive 2010/63/EU of the European Parliament and of the Council of 22 September 2010 on the Protection of Animals Used for Scientific Purposes. Off. J. Eur. Union.

[53] Zin S.M., Habib S., Yasid N.A., Ahmad S.A. (2018). A Review on microbiological degradation of 2,4-Dinitrophenol. J. Exp. Microbiol. Toxicol..

[54] Rifkind J.M., Araki K., Hadley E.C. (1983). The relationship between the osmotic fragility of human erythrocytes and cell age. Arch. Biochem. Biophys..

[55] Meng F., Alayash A.I. (2017). Determination of extinction coefficients of human hemoglobin in various redox states. Anal. Biochem..

[56] Dotsenko O.I. (2014). Acid-hemolytic stability of erythrocytes of intense erythropoiesis under conditions of low-frequency vibration. Visn. Dnipropetr. Univ. Ser. Biol. Ekol..

[57] Repina S.V., Repin N.V. (2008). Peculiarities of RBCs resistance to acid hemolysis in hibernating mammals. Bioelectrochem.

[58] Mikaelyan M.S., Shahinyan M.A., Parsadanyan M.A. (2023). Erythrocytes as a model for studying the effect of temperature on the resistance of cellular membranes. Proceeding Yerevan State Univ. Chem. Biol..

[59] Delgado A.V., González-Caballero F., Hunter R.J., L Koopal K., Lyklema J. (2007). Measurement and Interpretation of Electrokinetic Phenomena. J. Colloid Interface Sci..

[60] McLaughlin S. (1977). Electrostatic potentials at membrane-solution interfaces. Curr. Top. Membr. Transp..

[61] Freire J.M., Domingues M.M., Matos J., Melo M.N., Veiga A.S., Santos N.C., Castanho M.A.R.B. (2011). Using zeta potential measurements to quantify peptide partition to lipid membranes. Eur. Biophys. J..

[62] Cevc G. (1990). Membrane electrostatics. Biochim. Biophys. Acta.

[63] Doltchinkova V., Lozanova S., Rukova B., Nikolov R., Ivanova E., Roumenin C. (2023). Electrokinetic properties of healthy and β-thalassemia erythrocyte membranes under in vitro exposure to static magnetic field. Front. Chem..

[64] Israelashvili J.N., Israelashvili J.N. (2011). Electrostatic Forces between Surfaces and Liquids. Intermolecular and Surface Forces. Part III: Self-Assembling Structures and Biological Systems.

[65] Lepke S., Heberle J., Passow H., Bernhardt I., Ellory J.C. (2003). The Band 3 Protein: Anion Exchanger and Anion-Proton Cotransporter. Red Cell Membrane Transport in Health and Disease.

[66] Halliwell B., Gutteridge J.M.C., Halliwell B., Gutteridge J.M.C. (1999). Free Radicals in Biology and Medicine. Free Radicals in Biology and Medicine.

[67] Kanias T., Acker J.P. (2010). Mechanism of hemoglobin-induced cellular injury in desiccated red blood cells. Free Radic. Biol. Med..

[68] Mimnaugh E.G., Trush M.A., Bhatnagar M. (1985). Enhancement of reactive oxygen-dependent mitochondrial membrane lipid peroxidation by anticancer drug adriamycin. Biochem. Pharmacol..

[69] ZEISS ZEN (Blue Edition). www.zeiss.com/microscopy.

[70] Goss G.G., Adamia S., Galvez F. (2001). Peanut lectin binds to a subpopulation of mitochondria-rich cells in the rainbow trout gill epithelium. Am. J. Physiol. Regulatory Intergrative Comp. Physiol..

[71] Karabaliev M., Paarvanova B., Savova G., Tacheva B., Georgieva R. (2025). In Situ Monitoring of Morphology Changes and Oxygenation State of Human Erythrocytes During Surfactant-Induced Hemolysis. Cells.

[72] Paarvanova B., Tacheva B., Savova G., Karabaliev M., Georgieva R. (2023). Hemolysis by Saponin is Accelerated at Hypertonic Conditions. Molecules.

[73] Ivanov S., Galutzov B., Ratcheva-Kantcheva M., Markov M., Blank M. (1988). Radioprotective effects of ATP on in vitro gamma-irradiated human red blood cells. Electromagnetic Fields and Biomembranes.

[74] Hsu J.-P., Lin S.-H., Tseng S. (2003). Effect of membrane structure of human erythrocytes on its electrophoresis. Colloids Surf. B Biointerfaces.

[75] Zhang X., Lin Y., Xin J., Zhang Y., Yang K., Luo Y., Wang B. (2024). Red blood cells in biology and translational medicine: Natural vehicle inspires new biomedical applications. Theranostics.

[76] Kotyńska J., Petelska A.D., Szeremeta M., Niemcunowicz-Janica A., Figaszewski Z.A. (2012). Changes in surface-charge density of blood cells after sudden unexpected death. J. Membr. Biol..

[77] Huang Y.-X., Wu Z.-J., Mehrishi J., Huang B.-T., Chen X.-J., Zheng X.-J., Liu W.-J., Luo M. (2011). Human red blood cell aging: Correlative changes in surface charge and cell properties. J. Cell. Mol. Med..

[78] Adewoyin A.S., Adeyemi O., Davies N.O., Ogbenna A.A., Tombak A. (2019). Erythrocyte Morphology and Its Disorders. Erythrocyte.

[79] Pajic-Lijakovic I., Milivojevic M., Barshtein G., Gural A. (2025). The Mechanical Properties of Erythrocytes Are Influenced by the Conformational State of Albumin. Cells.

[80] Liao P.C., Pon L.A. (2024). Analysis of the mitochondria-associated degradation pathway (MAD) in yeast cells. Methods Enzymol..

[81] Navaro A., Boveris A. (2007). The mitochondrial energy transduction system and their aging process. Am. J. Physiol. Cell Physiol..

[82] Coca A., Garay R. (1988). Disturbances in Na^+^ transport systems induced by ethanol in human red blood cells. Alcohol. Clin. Exp. Res..

[83] Vani R., Anuska B.A., Christina R.M., Kavin P., Mohammed O., Inchara S., Kavvyasruthi U.J., Sadiya S., Sindhu H.S., Vani R. (2024). Band 3 Protein: A Critical Component of Erythrocyte. Red Blood Cells-Functions and Significance.

[84] Tosteson M.T., Holmes S.J., Razin M., Tosteson D.C. (1985). Melittin lysis of red cells. J. Membr. Biol..

[85] Gooz M., Maldonado E.N. (2023). Fluorescence microscopy imaging of mitochondrial metabolism in cancer cells. Front. Oncol..

[86] Doltchinkova V., Angelova P., Ugrinov K., Dimitrova I. (2004). Effect of bee venom melittin on biological membranes III. Electrokinetic properties of rat liver mitochondria. Annuaire de l’Universite de Sofia “St. Kliment Ohridski”.

[87] Pérez-Payá E., Dufourcq J., Braco L., Abad C. (1997). Structural characterization of the natural membrane-bound state of melittin: A fluorescence study of a dansylated analogue. Biochim. Biophys. Acta.

[88] Hall K., Lee T.-H., Aguilar M.-I. (2010). The role of electrostatic interactions in the membrane binding of melittin. J. Mol. Recognit..

[89] Lee A.G. (2003). Lipid-protein interactions in biological membranes: A structural perspective. Biochim. Biophys. Acta.

[90] Terwilliger T.C., Eisenberg D. (1982). The structure of melittin. II. Interpretation of the structure. J. Biol. Chem..

[91] Altenbach C., Froncisz W., Hyde J.S., Hubbell W. (1989). Conformation of spin-labeled melittin at membrane surfaces investigated by pulse saturation recovery and continuous wave power saturation electron paramagnetic resonance. Biophys. J..

[92] Juhaniewicz J., Sek S. (2016). Interaction of Melittin with Negatively Charged Lipid Bilayers Supported on Gold Electrodes. Electrochim. Acta.

[93] Duann X., Zou H., Yang J., Liu S., Xu T., Ding J. (2024). Melittin-incorporated nanomedicines for enhanced cancer immunotherapy. J. Control. Release.

